# Synthesis of Sulfur-35-Labeled Trisulfides and GYY-4137 as Donors of Radioactive Hydrogen Sulfide

**DOI:** 10.1021/acsomega.3c03258

**Published:** 2023-07-19

**Authors:** Eric M. Brown, James P. Grace, Nimesh P. R. Ranasinghe Arachchige, Ned B. Bowden

**Affiliations:** W425 Chemistry Building, Department of Chemistry, University of Iowa, Iowa City, Iowa 52242 United States

## Abstract

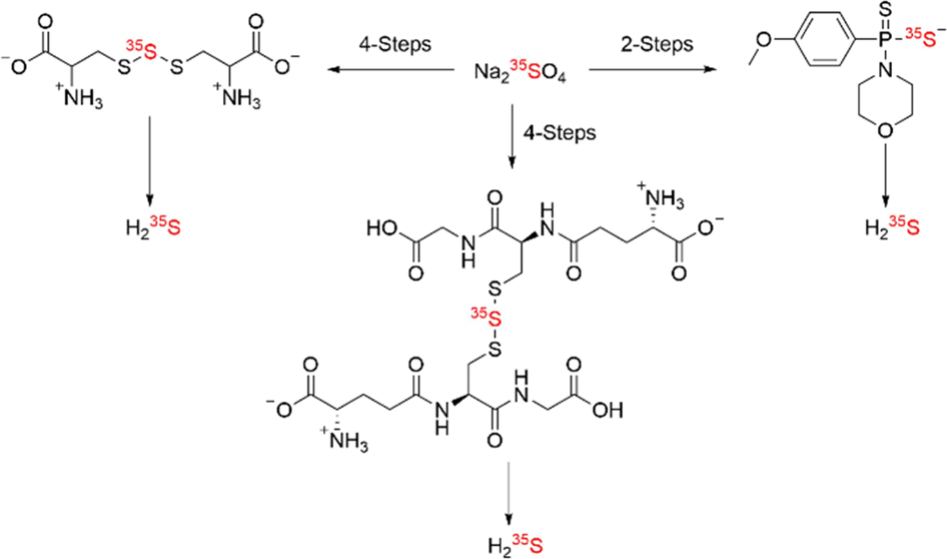

Hydrogen sulfide has emerged as a key gasotransmitter in humans and in plants, and the addition of exogenous hydrogen sulfide has many beneficial effects *in vivo* and *in vitro*. A challenge in investigating the effect of exogenous hydrogen sulfide is tracking the location of exogenous hydrogen sulfide on an organism and cellular level. In this article, we report the synthesis of three key chemicals (cysteine trisulfide, glutathione trisulfide, and GYY-4137) that release radiolabeled ^35^S as hydrogen sulfide. The synthesis started with the reduction of Na_2_^35^SO_4_ mixed with Na_2_SO_4_ to generate hydrogen sulfide gas that was trapped with aq NaOH to yield radiolabeled Na_2_S. The Na_2_S was converted in one step to GYY-4137 at 65% yield. It was also converted to bis(tributyltin) sulfide that readily reacted with *N*-bromophthalimide to yield a monosulfur transfer reagent. Trisulfides were synthesized by reaction with the monosulfur transfer reagent and the corresponding thiols. The levels of radioactivity of the final products could be varied on a per gram basis to alter the radioactivity for applications that require different loadings of hydrogen sulfide donors.

## Introduction

Hydrogen sulfide (H_2_S) is a widely studied gasotransmitter in medicinal biochemistry and is responsible for modulating a host of physiological pathways affecting cardiovascular, endocrine, and neurological systems.^1−5^ Although H_2_S is present in nM concentrations in human cells, it can be dosed at sub-symptomatic concentrations to elicit a variety of beneficial effects, including smooth muscle relaxation, angiogenesis, neuromodulation, and antioxidative properties.^6−12^ It has been shown to help treat cardiovascular diseases, acute lung injury, neurological aging, and some cancers in animal models, although dosing and timing of administration appear to be critical and complex.^13−19^ Although some physiological effects of H_2_S have been well studied, much is unknown about its role in these physiological pathways and which organs are most affected.

Recent work showed that the addition of exogenous H_2_S led to the formation of persulfides (RSSH) on hundreds to thousands of proteins and that these persulfides are important for understanding how H_2_S affects dozens of enzymatic cycles.^20−23^ Furthermore, H_2_S has a moderate p*K*_a_ of 7.0 and is nonpolar, so it rapidly permeates cell membranes without the aid of a transport protein.^24,25^ A challenge in studying the effect of the addition of exogenous H_2_S in biological systems is that its concentration *in vivo* is carefully controlled at the nM level by conversion of excess H_2_S to persulfides, trisulfides, and other chemicals.^26^ It is unknown where exogenous H_2_S is trafficked *in vivo* and which enzymes it preferentially reacts with. Although the addition of exogenous H_2_S correlates with the up- or downregulation of hundreds of proteins and likely leads to persulfidation, these effects are observed without knowledge of where exogenous H_2_S is located within cells or how rapidly it moves between cells.^21,25,27−30^

**Figure 1:**
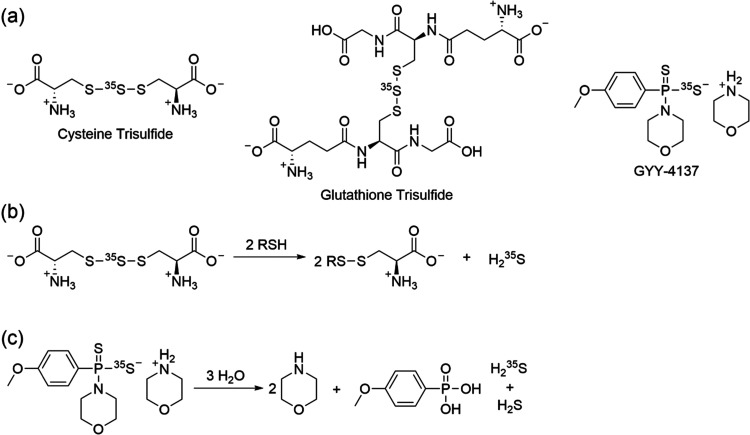
(a) Structures of ^35^S-labeled cysteine, glutathione trisulfide, and GYY-4137 are shown. (b) Degradation pathway of S-35 cysteine trisulfide in the presence of a thiol leading to the formation of H_2_^35^S. (c) Structure and degradation pathway of S-35 GYY-4137 in the presence of water leading to the formation of H_2_^35^S.

To address these challenges, we synthesized two natural trisulfides (glutathione trisulfide and cysteine trisulfide) and GYY-4137 with radioactive sulfur-35 (Figure 1). Trisulfides of glutathione and cysteine are found in μM concentrations *in vivo* and react with thiols to yield persulfides and H_2_S. These trisulfides are important sources of sulfane sulfur *in vivo* and are believed to be reservoirs of H_2_S.^31−33^ GYY-4137 is a widely studied H_2_S donor that releases H_2_S in a slow, controlled rate over days to weeks *in vivo*.^34−37^ Chemicals that slowly release H_2_S are widely used due to the difficulties of using gaseous or aqueous solutions of H_2_S that are easily generated by the addition of solid NaSH to water. One drawback of using aq H_2_S is its low boiling point (−60 °C) and high toxicity at ppm levels. The application of aqueous solutions of H_2_S leads to unrealistically high concentrations of H_2_S that rapidly decrease over minutes to hours, which necessitates their repeated application.^13,38^ To address this problem, numerous H_2_S donors that release H_2_S by hydrolysis, light, reaction with thiols, or pH have been developed.^36,39−42^ Trisulfides and GYY-4137 are important examples of these H_2_S donors.^36,43^

The radioactive isotope of sulfur is ^35^S, which is a low energy β emitter with a maximum β energy of 0.167 MeV and a relatively long half-life (*t*_1/2_ = 87.5 days).^44^ β-Decay of the sulfur atom yields _17_^35^Cl with the release of an electron and an anti-neutrino eq 1.^45^
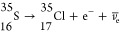
1

γ Decay is not observed in ^35^S, which significantly limits biological damage compared to isotopes that release high-energy photons of γ radiation. β-Decay in small amounts is safe with low permeation through simple materials.^46^ For instance, the radiation is adsorbed by ∼2 feet of air or by a thin layer of dead skin, so no radiation protective equipment is necessary. Additionally, the long half-life allows time for synthetic transformation of radioactive sulfur and applications without the need for further enrichment. Sulfur-35 has been tracked *in vivo* by incorporation in cysteine or by synthesis of chemicals from radioactive sulfates.^47,48^ Sulfur-35-enriched Na_2_^35^SO_4_ is the only commercially available starting material outside of ^35^S enriched-amino acids for the synthesis of chemicals. The Na_2_^35^SO_4_ is sold at a radioactive level of 1 mCi (∼0.01 μg of ^35^S), but this level is orders of magnitude too high for liquid scintillation and autoradiographic techniques that detect ^35^S at levels of 2.0–2.8 μCi.

This article reports the rapid (5-day) synthesis of trisulfides and GYY-4137 using Na_2_^35^SO_4_. These chemicals will release H_2_^35^S in the presence of thiols (trisulfides) and water (GYY-4137). The synthetic routes are simple and can be used to produce 1–10 g of each of the final products with known and predictable amounts of radiation. These chemicals can be used to further investigate their location and transport *in vivo* and to determine where H_2_^35^S partitions within cells.

## Results and Discussion

### Reduction of Sulfate to Sulfide

The synthetic pathways to trisulfides and GYY-4137 were investigated using nonradioactive samples for cost and safety considerations. After the pathways were optimized and repeated several times, the radioactive sulfate was used. The possibility of leakage of H_2_S from all reactions was prevented by using aq NaOH and bleach traps, as described in the Supporting Information. Furthermore, lead acetate strips that turn from white to black in the presence of H_2_S were used at the final vent of each reaction and near selected joints in the glassware to ensure that no H_2_S was being released.

**Figure 2:**
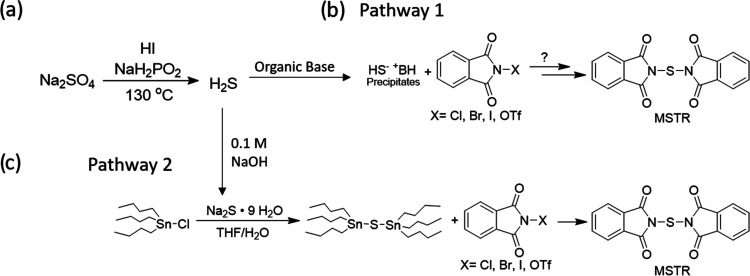
(a) Reduction from sodium sulfate to H_2_S using hydriodic acid (HI) and sodium hypophosphite (NaH_2_PO_2_) at 130 °C is shown. (b) In pathway 1, the H_2_S would be trapped with an organic base in an organic solvent, followed by a reaction with *N*-halophthalimide to yield the MSTR. (c) In pathway 2, the H_2_S would be trapped with aq NaOH, and this would be used to synthesize bis(tributyltin) sulfide. The bis(tributyltin) sulfide would be reacted to yield the MSTR.

The first step in the synthesis was the reduction of sulfate to sulfide, as shown in Figure 2a. The radioactivity of ∼0.01 μg of Na_2_^35^SO_4_ in 1 mL of water is 1 mCi with a specific activity of 1600 Ci/mmol, which was over three orders of magnitude too radioactive for sensors of ^35^S that detect μCi levels of ^35^S and a minute amount of sulfate to react. In reactions with Na_2_^35^SO_4_, an additional amount of 0.5–1.0 g of Na_2_SO_4_ was added to the reaction. The addition of nonradioactive sulfate allowed the reaction to proceed with a larger, easier to handle quantity of sulfate and ultimately produced enough sulfide for further reactions and purification. The nonradioactive sulfate also diluted the ^35^S to levels that were needed in the end products. For instance, in prior studies using GYY-4137 to deliver H_2_S, the amounts of GYY-4137 that were used to generate sufficient H_2_S to have the desired response ranged from 1 to 100 mg.^34,49,50^ Diluting Na_2_^35^SO_4_ with nonradioactive sulfate allowed us to tailor the radioactivity of the final samples of GYY-4137 and trisulfides to possess desired and easy to measure μCi levels of ^35^S in loadings of 1 to 100 mg of GYY-4137.

The reduction reaction used a variation of the Nagai reduction, which employed hydriodic acid (57% HI) and sodium hypophosphite (NaH_2_PO_2_) at elevated temperatures to yield H_2_S from sodium sulfate in quantitative yields (Figure 2a).^51^ The reduction was carried out under N_2_ gas, and the generated H_2_S was bubbled to a second flask to trap it as a salt (see Figure S1 for a schematic of the glassware that was used in this reaction).

The reduction yielded an aqueous, acidic solution of H_2_S, which needed to be trapped as a salt. Two pathways to trap H_2_S to ultimately synthesize a monosulfur transfer reagent (MSTR) were investigated, as shown in Figure 2b,c. The MSTR was an important intermediate because prior work by us showed that it reacted with thiols to produce trisulfides in high yields. In one method, the H_2_S generated from the reduction of sulfate was bubbled into an organic solvent with a base present to attempt to yield a solid precipitate of HS^–^ X^+^. Solvents such as hexanes, tetrahydrofuran (THF), DMF, and pure NE*t*_3_ were investigated with bases such as NE*t*_3_ and KO*^t^*Bu (Table S1). In none of these reactions was a precipitate observed. Although the sulfide could be converted into a base in organic solvents, due to safety issues of handling ^35^S, it was decided to not remove the organic solvent under vacuum. A second method to isolate H_2_S was investigated by using NaOH dissolved in water in a concentration of 0.10 M at a pH of 13. The first p*K*_a_ of H_2_S is 7.0, and the second p*K*_a_ is approximately 10, so it was hypothesized that a basic solution of NaOH in water would effectively trap it as Na_2_S.

**Figure 3:**
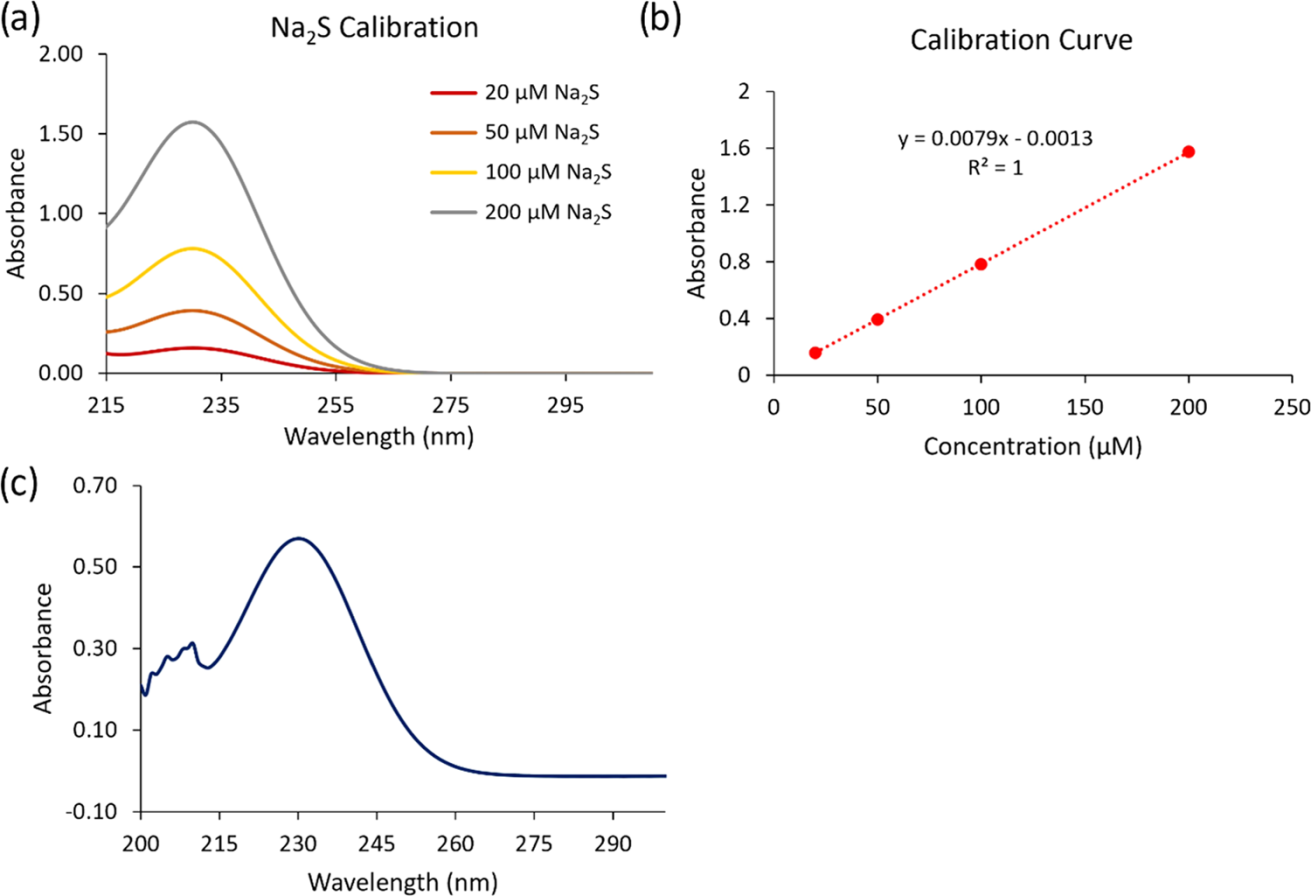
(a) UV–vis spectroscopy of prepared solutions of Na_2_S·9H_2_O at known concentrations (0, 20, 50, 100, and 200 μM) was plotted. (b) Calibration curve generated from the peak at 230 nm for samples of Na_2_S·9H_2_O is shown. (c) The UV–vis spectroscopy of the Na_2_S obtained after reduction of sulfate is shown.

To investigate the ability of 0.10 M NaOH to trap H_2_S, ultraviolet–visible (UV–vis) spectroscopy was used to measure the concentration of Na_2_S. First, a calibration curve of the UV peak at 230 nm for Na_2_S was obtained using aqueous solutions in 0.10 M NaOH (Figure 3a,b). Next, reactions were completed to measure if the glassware and tubing used for the reduction of sulfate to sulfide would trap H_2_S. A flask was charged with a known amount of sodium sulfide nonahydrate (Na_2_S·9H_2_O, 0.624 g, 2.6 mmol) and connected to the glassware used to reduce Na_2_SO_4_ and trap the resulting H_2_S in aq NaOH. An excess of HCl was slowly dripped onto the sodium sulfide nonahydrate, and N_2_ as a carrier gas was used at a pressure of 3 psi to deliver the H_2_S gas to a bubbler within the aq NaOH. The concentration of Na_2_S was measured and found to be 76 mM, which was an 88% trapping yield. After confirmation that the H_2_S was trapped in high yield, the same trapping technique was used to trap H_2_S generated by the reduction of sulfate using HI/NaH_2_PO_2_. UV–vis spectroscopy experiments showed that the sulfide was trapped by aq NaOH with a yield of 95% (Figure 3c). This method demonstrated that the sulfate could be reduced and trapped as sulfide in high yields with simple glassware.

### Conversion of Aqueous Sulfide to Bis(tributyltin) Sulfide

The use of aq NaOH to trap sulfide presented a challenge to synthesize the MSTR by reaction of the Na_2_S with *N*-halophthalimide. The *N*-halophthalimides were insoluble in water, and biphasic reactions with *N*-halophthalimide and aqueous sulfide were unsuccessful. The Na_2_S was dissolved in water, but it was decided not to evaporate the water due to the potential release of ^35^S.

The aqueous sulfide solution was reacted with stoichiometric amounts of tributyltin chloride (2.0 equiv per equivalent of Na_2_S) for 4 h at 65 °C. Bis(tributyltin) sulfide was isolated in a low yield (48%) compared to reported literature values (90%), and tributyltin chloride was present as an impurity. The low yield was due to the lower concentration of Na_2_S in these experiments (0.100 M) compared to concentrations in the literature (0.750 M) and because prior work used an excess of sulfide. To address these concerns, the trapped sulfide was supplemented with Na_2_S·9H_2_O to yield a final concentration of 0.2 M, and a slight excess of tributyltin chloride (2.1 equiv) was used. The reaction was allowed to proceed for 16 h to ensure it was complete; longer reactions did not increase the yield. These reaction conditions yielded bis(tributyltin) sulfide in 72% purity with an isolated yield of 69%. The impure product was used directly without purification.

### Conversion of Bis(tributyltin) Sulfide to MSTR

**Figure 4:**
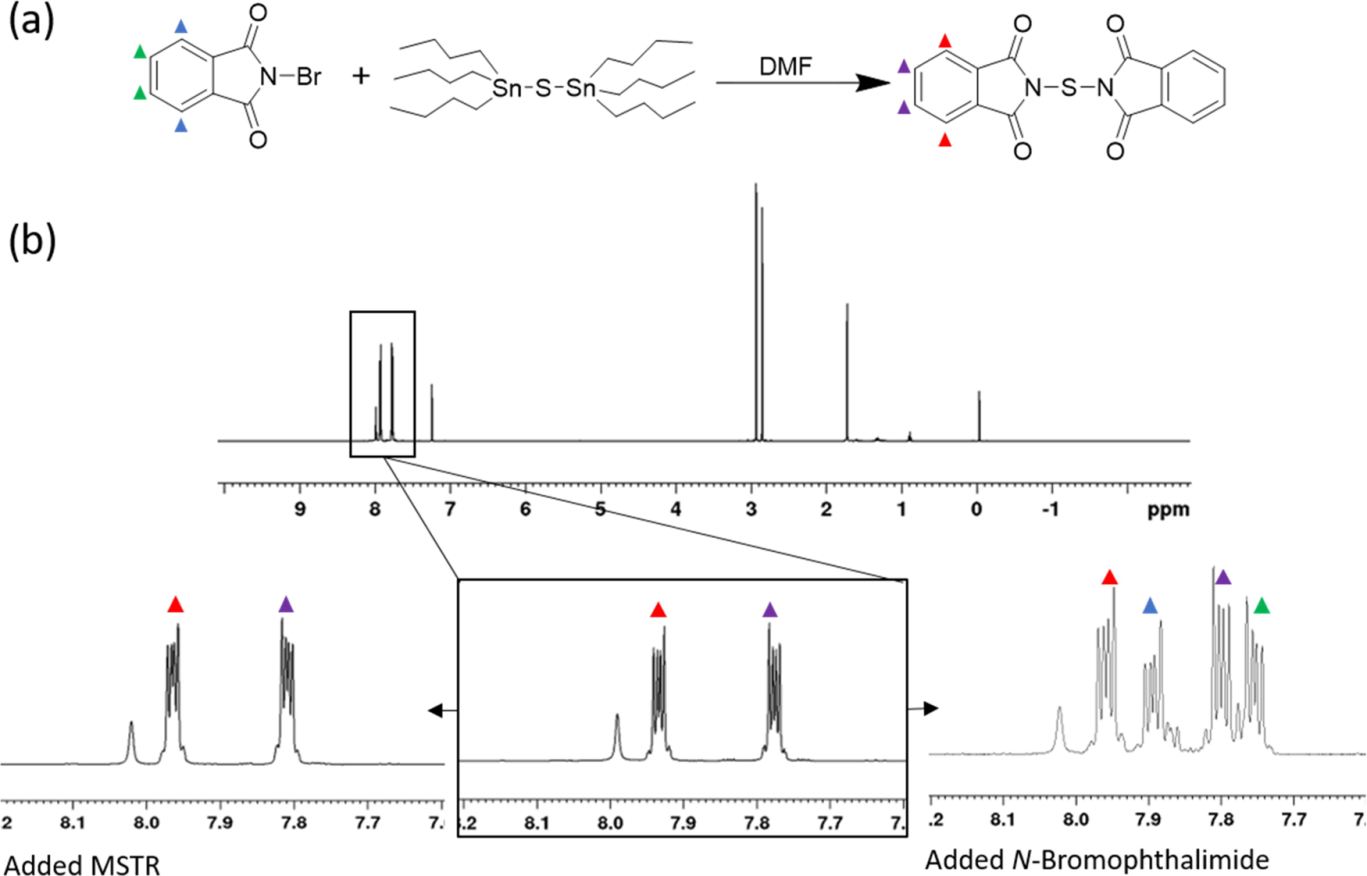
(a) Reaction of NBP and bis(tributyltin) sulfide in a concentrated solution of DMF yielded the MSTR. Colored triangles represent aromatic hydrogen shift assignments for *N*-bromophthalimide and MSTR. (b) ^1^H NMR spectrum of crude MSTR with expanded aromatic region is shown. The NMR solution spiked with pure MSTR revealed no change in peak shifts or additional peaks. The NMR solution spiked with *N*-bromophthalimide revealed a new set of downfield peaks indicating a complete reaction.

The reaction of bis(tributyltin) sulfide with *N*-bromophthalimide was investigated in DMF. This solvent was selected because the MSTR is insoluble in DMF, so purification by filtration would simplify the removal of impurities. To investigate if bis(tributyltin) sulfide could generate MSTR, it was added dropwise over 5 min to excess *N*-bromophthalimide in DMF. The reaction was stirred for 16 h, and the precipitate was isolated by filtration. The white solid was analyzed *via*^1^H NMR spectroscopy, which showed that MSTR had been successfully synthesized, albeit in a low yield (20%). The product was confirmed by spiking the isolated solid with known samples of pure MSTR and *N*-bromophthalimide. Downfield chemical shifts from *N*-bromophthalimide can be observed from the aromatic hydrogens (Figure 4b). Furthermore, spiking the sample with a disulfide transfer reagent demonstrated that the product was not the disulfide transfer reagent.

A series of experiments were completed to optimize this reaction to increase the yield of MSTR (Table 1). Completing the reaction in DMF at varying temperatures did not improve the initial yield of 20%. Cooling the reaction to −60 °C yielded no MSTR. Surprisingly, changing the solvent also yielded no MSTR. Using acetonitrile, DCM, MeOH, and hexanes as solvents each afforded phthalimide and an unknown byproduct with ^1^H NMR shifts in the aromatic region. Both *N*-chlorophthalimide and *N*-iodophthalimide yielded very little MSTR. Moving forward with DMF and *N*-bromophthalimide, additives were investigated to increase the yield. Bis(tributyltin) sulfide is often used in combination with fluoride sources to increase the nucleophilicity of sulfide. Therefore, cesium fluoride was added with catalytic 18-crown-6, but no precipitate formed in the reaction flask. Tetrabutylammonium fluoride (TBAF) was also used as a fluoride source, but no precipitate formed. The next additive attempted was phthalimide based on experiments conducted by Hunter et al., where 1-chlorobenzotriazole, thiols, and benzotriazole were reacted (Figure S2). In this prior work, thiols were converted to their respective SCl derivatives by 1-chlorobenzotriazole, which reacted with benzotriazole to form a less reactive electrophilic sulfur source.^52^ Using this same technique, we hypothesized using phthalimide as an additive would increase the yield of MSTR. The yield was increased to 35% when phthalimide was added, which further increased to 53% upon increasing the reaction time to 40 h. No increase in yield was observed after 40 h. The MSTR was isolated as a single product by filtration.

**Table 1:** Optimization of the Reaction between *N*-Bromophthalimide and Bis(tributyltin) Sulfide to form MSTR

phthalimide derivative used	solvent	additive	temperature (C)	time (h)	MSTR yield (%)
*N*-bromophthalimide	DMF		RT	16	20
*N*-bromophthalimide	DMF		RT	40	20
*N*-bromophthalimide	DMF		0	16	18
*N*-bromophthalimide	DMF		–60	8	0
*N*-bromophthalimide	DMF		50	16	15
*N*-bromophthalimide	MeCN		RT	16	0
*N*-bromophthalimide	DCM		RT	16	0
*N*-bromophthalimide	DCM		0	16	0
*N*-bromophthalimide	MeOH		RT	16	0
*N*-bromophthalimide	hexanes		RT	16	0
*N*-bromophthalimide	formamide		RT	16	0
*N*-bromophthalimide	DMA		RT	16	0
*N*-chlorophthalimide	DMF		RT	16	<1
*N*-iodophthalimide	DMF		RT	16	0
*N*-bromophthalimide	DMF/EtOAc (5:1)		RT	16	20
*N*-bromophthalimide	DMF	18-crown-6, CsF	RT	16	0
*N*-bromophthalimide	DMF	TBAF	RT	16	0
*N*-bromophthalimide	DMF	phthalimide	RT	16	35
*N*-bromophthalimide	DMF	phthalimide	RT	48	53

### Synthesis of Trisulfides from MSTR

**Figure 5:**
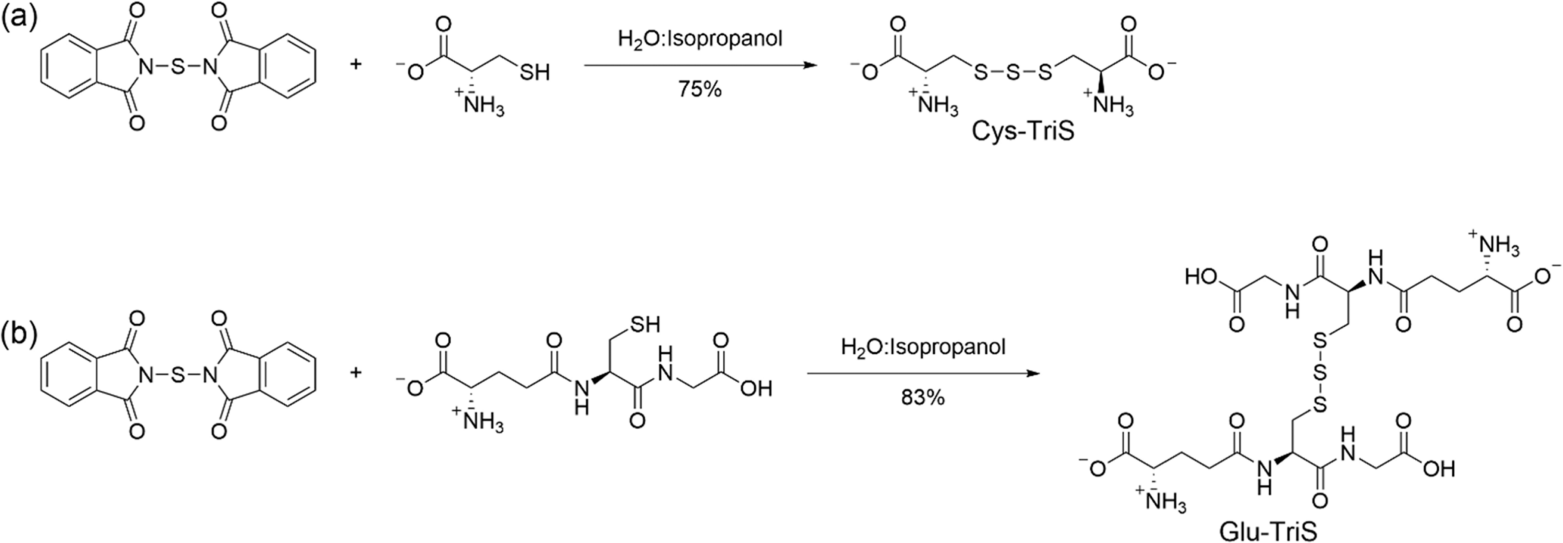
MSTR was used to synthesize (a) l-cysteine trisulfide (Cys-TriS) and (b) glutathione trisulfide (Glu-TriS).

The next step in the synthesis was reaction of the MSTR with l-cysteine and glutathione to yield the trisulfides. In prior work, we used the MSTR to synthesize trisulfides from l-cysteine and glutathione, but these reactions used an excess of the MSTR.^43^ The synthesis was altered to use stoichiometric amounts of MSTR because this compound possessed the radioactive sulfur. To achieve a high yield, a syringe pump was used to control and slow the rate of addition of the amino acid to MSTR. A rate of 15 mL/h afforded cysteine trisulfide in a 75% yield and glutathione trisulfide in an 83% yield using stoichiometric amounts of MSTR (Figure 5).

This reaction sequence was completed using ^35^S, as described in detail in the Supporting Information. Starting from 0.005 μg of radiolabeled sulfate, 0.35 g of Cys-TriS and 0.63 g of Glu-TriS were isolated. The overall yield of Cys-TriS was 22% from sulfate, and it was 23% for Glu-TriS. The trisulfides were characterized by liquid scintillation, which measured the radioactivity of the Cys-TriS as 21 μCi per 100 mg and Glu-TriS as 34 μCi per 100 mg on the day that these syntheses were completed.

### Synthesis of GYY-4137 from Aqueous Sulfide

**Figure 6:**
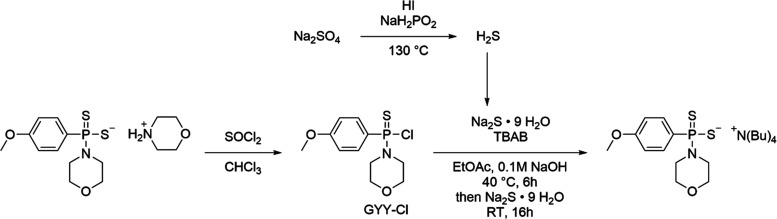
Synthetic pathway for the synthesis of radioactive GYY-4137 from sodium sulfate and GYY-Cl is shown.

The synthesis of GYY-4137 from Na_2_S was brief and required only two steps (Figure 6). First, the thiophosphoryl chloride derivative of GYY-4137 (GYY-Cl) was easily obtained upon reaction of GYY-4137 with thionyl chloride in chloroform. This reaction proceeded to a yield of 66% and was purified by column chromatography.

Next, the reaction of GYY-Cl with Na_2_S was investigated. In the synthesis of radiolabeled trisulfides, the sulfate was reduced to sulfide and trapped in 0.1 M aq NaOH; this solution was used as the starting material. Initially, GYY-Cl was reacted with Na_2_S using a homogenous ethanol/water solvent mixture, but the yields were poor, and numerous byproducts were observed. Next, aqueous Na_2_S was diluted with THF, but again, reactions with GYY-Cl gave poor yields. A biphasic solvent mixture consisting of CHCl_3_/water was investigated with tetrabutylammonium bromide as a phase transfer catalyst, and the ^31^P NMR yield was 65% after 1 day. The only observed impurity was the GYY-Cl starting material, and increasing the reaction time to 3 days increased the yield to 73%, but multiple impurities were observed, likely due to degradation of GYY-Cl or GYY-4137. The organic solvent was replaced with EtOAc, which did not give any improvement in yield but reduced the amount of impurities present. Using the same reaction conditions but lowering the temperature to 0 °C did not give any improvement in yield. Heating the reaction mixture to 40 °C increased the yield to 86% after 1 day but also gave multiple impurities, including unreacted GYY-Cl. Reducing the time to 6 h lowered the yield to 70%, but the only observed impurity was the GYY-Cl starting material. To consume all of the starting material, an additional one equivalent of Na_2_S·9H_2_O was added after 6 h of stirring at 40 °C, and the mixture was stirred for an additional 16 h at room temperature to give the tetrabutylammonium salt of GYY-4137 in a 66% isolated yield (Figure 6).

This reaction sequence was completed using ^35^S, as described in detail in the Supporting Information. Starting from 0.005 μg of radiolabeled sulfate, 1.8 g of GYY-4137 was isolated in 65% yield from GYY-Cl. GYY-4137 was characterized by liquid scintillation, which measured the radioactivity of GYY-4137 as 30 μCi per 100 mg on the day that the synthesis was completed.

## Conclusions

A 4-step synthetic pathway was developed to synthesize radioactive cysteine trisulfide and glutathione trisulfide from radioactive sodium sulfate in 22 and 23% overall yields, respectively. A key step in this synthesis was the synthesis of a MSTR in DMF. The MSTR was insoluble in DMF, which simplified purification from the numerous impurities that were carried from the prior reaction. The synthesis can be completed in 5 days or less from a mixture of radiolabeled and nonradioactive sulfate. The 2-step synthesis of radioactive GYY-4137 was also completed in a yield of 65% starting from GYY-Cl. These syntheses are versatile, and starting from commercially available microgram quantities of Na_2_^35^SO_4_, multigram quantities of trisulfides or GYY-4137 could be synthesized to possess varied and controlled levels of radioactivity. The concentrations of ^35^S in the final products were easily varied by altering the ratio of nonradioactive sulfate to radioactive sulfide in the reduction to sulfide. Controlling the level of radioactivity within the final products is important for future work where milligram quantities of trisulfides or GYY-4137 will be needed to provoke a response due to H_2_S, and the released H_2_^35^S will be at desired levels of μCi of radiation that can be detected using liquid scintillation or autoradiography.

## Experimental Section

### Materials and Methods

All solvents were purchased from Sigma-Aldrich or Fischer Scientific and dried prior to use with anhydrous MgSO_4_. ^1^H, ^13^C, and ^31^P NMR spectra were recorded on either an Avance 300 or 75 MHz NMR instrument or an Avance 400 and 100 MHz NMR instrument. Ultraviolet–visible spectra were collected on an Agilent Cary 5000 UV–vis/NIR Spectrophotometer using a single-front method baselined with 0.1 M NaOH in Optima grade water (200–400 nm). All chemicals were purchased from Sigma-Aldrich or Acros Organics and used without further purification unless otherwise stated. A 1 mCi sample of Na_2_^35^SO_4_ in 1 mL of water with a specific activity of 1600 Ci/mmol was purchased from PerkinElmer. All yields are isolated yields unless reported otherwise.

### Reduction of Na_2_SO_4_ to H_2_S and Trapping H_2_S

The reduction of sulfate to sulfide was adapted from a known literature procedure.^51^ Sodium sulfate (Na_2_SO_4_) (493 mg) was added to a pre-dried Schlenk flask with a PTFE N_2_ gas bubbler. The pressure of the gas was at 3 psi, and the Schlenk flask was connected with tygon tubing to five other flasks in sequence. A schematic of the glassware is shown in Figure S1. First, a reducing solution consisting of 2.0 g of NaH_2_PO_2_ dissolved in 15 mL of 57% HI was added *via* a syringe to the flask with sodium sulfate. The reaction was heated to 130 °C for 3 h to fully reduce sulfate chemicals to sulfides. The gas was passed to two other flasks with 20 mL of Milli-Q Optima grade water to trap any unwanted byproducts. The gas was bubbled through the water in each flask, and both solutions became turbid during the reaction. Next, the gas was bubbled into a flask with 40 mL of aq 0.1 M NaOH to collect the H_2_S as Na_2_S. Finally, the gas was bubbled through a flask with 60 mL of a 2.0 M NaOH solution and then through a 6% NaOCl trap (60 mL) before venting through a charcoal trap. The concentration of trapped NaSH/Na_2_S was determined by UV–vis spectroscopy (82.4 mM, 95% yield).

### Synthesis of Bis(tributyltin) Sulfide from Aqueous Na_2_S

The synthesis of bis(tributyltin) sulfide was adapted from a known literature procedure.^53^ To a stirred solution of Na_2_S (65.5 mM, 2.62 mmol) from the previous step was added an additional amount of Na_2_S·9H_2_O (1.41 g, 5.87 mmol) to reach the desired sulfide concentration (0.2 M). In a separate flask, tributyltin chloride (5.75 g, 17.8 mmol) was dissolved in 70 mL of THF, and the solution was added to the reaction flask containing sulfide. The flask was washed with 45 mL of THF, and 10 mL of water and the washings were added to the reaction flask. The reaction was refluxed at 65 °C for 16 h while vigorously stirring, and then the product was concentrated under reduced pressure to remove THF. The aqueous phase was extracted with Et_2_O (3 × 20 mL), and the combined organic layers were dried over MgSO_4_ and concentrated under reduced pressure to yield bis(tributyltin) sulfide (3.67 g, 69%). Leftover starting material remained and was removed in the next step. ^1^H NMR (400 MHz, CDCl_3_) δ 1.52–1.66 (m, starting material and product, 12H), 1.27–1.38 (m, starting material and product, 12H), 1.06–1.10 (m, 12H), 0.90 (t, starting material and product, *J* = 7.1 Hz, 18H). ^13^C NMR (100 MHz, CDCl_3_) δ 28.81, 27.30, 15.99, 13.78. HRMS: calculated for C_12_H_27_Sn^+^: 291.1135; found: 291.1127

### Synthesis of 2,2′-Thiobis(isoindoline-1,3-dione) (MSTR)

To a stirred solution of phthalimide (4.95 g, 33.7 mmol) and bis(tributyltin) sulfide (7.71 g, 7.70 mmol) in DMF (40 mL) was added a solution of *N*-bromophthalimide (6.80 g, 30.5 mmol) in DMF (16 mL) dropwise over 5 min. Stirring was continued for an additional 48 h. The precipitate was collected *via* vacuum filtration. White solid was washed with 30 mL of toluene. The solid was dried fully under high vacuum to yield a fluffy white solid (1.33 g, 53%). ^1^H NMR (400 MHz, CDCl_3_) δ 7.94–7.96 (m, 4H), 7.78–7.80 (m, 4H). ^13^C NMR (100 MHz, CDCl_3_) δ 166.2, 135.2, 131.6, 124.7. HRMS: calcd for C_16_H_8_N_2_SO_4_H^+^: 325.0283; found: 325.0277.

### Synthesis of Cysteine Trisulfide (Cys-TriS)

The synthesis of Cys-TriS was adapted from a known literature procedure.^43^ To a solution of 2,2′-thiobis(isoindoline-1,3-dione) (0.912 g, 2.8 mmol) in isopropanol (8 mL), cysteine (0.340 g 2.8 mmol) dissolved in water (8 mL) was added *via* a syringe pump with a flow rate of 15 mL h^–1^. After stirring for an additional 12 h, the crude product was collected by vacuum filtration and washed with acetone and dichloromethane to yield the purified product as a white solid (0.724 g 75%). ^1^H NMR (400 MHz, D_2_O) δ 4.12 (dd, *J* = 8.0, 4.4 Hz, 2H), 3.52 (dd, *J* = 15.0, 4.2 Hz, 2H), 3.31 (dd, *J* = 15.0, 8.2 Hz, 2H). ^13^C NMR (100 MHz, D_2_O) δ 170.6, 51.9, 37.1. HRMS: calcd for C_6_H_12_N_2_O_4_S_3_H^+^, 273.0037; found: 273.0033.

### Synthesis of Glutathione Trisulfide (Glu-TriS)

The synthesis of Glu-TriS was adapted from a known literature procedure.^43^ To a solution of 2,2′-thiobis(isoindoline-1,3-dione) (0.215 g, 0.66 mmol) in isopropanol (5 mL), glutathione (0.396 g 1.3 mmol) dissolved in water (5 mL) was added *via* a syringe pump with a flow rate of 15 mL h^–1^. After stirring for an additional 30 min, the crude product was collected by vacuum filtration and washed with acetone and dichloromethane to give the purified product as a white solid (0.344 g, 83%). ^1^H NMR (400 MHz, D_2_O) δ 3.88 (s, 4H), 3.77 (t, *J* = 6.4 Hz, 2H), 3.42 (dd, *J* = 14.4, 4.8 Hz, 2H), 3.18 (dd, *J* = 14.6, 9.0 Hz, 2H), 2.50 (td, *J* = 7.8, 3.6 Hz, 4H), 2.12 (q, *J* = 7.5 Hz, 4H). HRMS: calcd for C_20_H_31_N_6_O_12_S_3_^–^, 643.1162; found: 643.1182.

### Synthesis of 4-Methoxyphenyl(morpholino)phosphinothioic Chloride (GYY-Cl)

To a solution of GYY-4137 (4.20 g, 11.2 mmol) in chloroform (11 mL), SOCl_2_ (2.50 mL, 34.5 mmol) was added dropwise over 5 min. The reaction was kept under an N_2_ atmosphere and stirred at room temperature for 16 h. The chloroform was removed under reduced pressure, and the crude yellow solid was purified by column chromatography using 98/2 dichloromethane/hexanes. The solvent was removed under reduced pressure to yield a white solid (2.15 g, 66%). ^1^H NMR (400 MHz, CDCl_3_) δ 7.86 (dd, *J* = 14.8, 8.9 Hz, 2H), 6.99 (dd, *J* = 8.9, 5.1 Hz, 2H), 3.87 (s, 3H), 3.68–3.70 (m, 4H), 3.39–3.44 (m, 2H), 3.02–3.08 (m, 2H). ^13^C NMR (100 MHz, CDCl_3_) δ 163.2, 132.9, 114.5, 66.6, 55.7, 45.5. ^31^P NMR (100 MHz, CDCl_3_) δ 85.6. HRMS: calcd for C_11_H_15_ClNO_2_PSH^+^, 292.0327; found, 292.0318.

### Synthesis of GYY-4137 from GYY-Cl

To a solution of GYY-Cl (0.907 g, 3.11 mmol) in ethyl acetate (25 mL), Na_2_S·9H_2_O (0.745 g, 3.10 mmol) and tetrabutylammonium bromide (1.90 g, 5.90 mmol) dissolved in 0.1 M NaOH (40 mL) were added. Two layers were formed. The solution was stirred at 40 °C for 6 h and monitored by ^31^P NMR spectroscopy. The reaction was 70% complete after 6 h, with the remainder as unreacted GYY-Cl. Additional Na_2_S·9H_2_O (1.03 g, 4.30 mmol) was added, and the reaction was stirred at room temperature for an additional 16 h. The ethyl acetate layer was extracted, and the aqueous layer was washed 3× with 20 mL of ethyl acetate. The organic layers were combined and dried over Na_2_SO_4_ and concentrated under reduced pressure to yield the tetrabutylammonium salt of GYY-4137 as an off-white solid. (1.07 g, 66%) ^1^H NMR (400 MHz, CDCl_3_) 8.15–8.20 (m, 2H), 6.80–6.83 (m, 2H), 3.80 (s, 3H), 3.61–3.63 (m, 4H), 3.32–3.36 (m, 8H), 3.01–3.03 (m, 4H), 1.59–1.67 (m, 8H), 1.36–1.45 (m, 8H), 0.97 (t, *J* = 7.3 Hz, 12H). ^13^C NMR (100 MHz, CDCl_3_) ^31^P NMR (100 MHz, CDCl_3_) 89.8. HRMS: calcd for C_11_H_15_NO_2_PS_2_, 282.0281; found, 288.0288.

### Calibration Curve for Quantification of Trapped Na_2_S by UV–vis Spectroscopy

Sodium sulfide nonahydrate (Na_2_S·9H_2_O) standards were prepared at known concentrations (0, 20, 50, 100, 200, and 500 μM) in 0.1 M NaOH using Optima grade H_2_O. All spectra were recorded in quartz cuvettes. Absorption data at 230 nm was plotted in Excel to generate a calibration curve.

## Supporting Information Available

^1^H and ^13^C NMR spectra of chemicals; description of synthesis of radiolabeled chemicals, description of the traps used to trap hydrogen sulfide (PDF)
