# Supplementary material for: Synthesis of Sulfur-35-Labeled Trisulfides and GYY-4137 as Donors of Radioactive Hydrogen Sulfide
Source: ACS Omega. 2023 Jul 19;8(30):27576–84. doi: 10.1021/acsomega.3c03258 (PMC10399151; doi:10.1021/acsomega.3c03258)
Supplement: Supplementary file 1 — ao3c03258_si_001.pdf [file ao3c03258_si_001.pdf]

Synthesis of Sulfur-35 Labeled Trisulfides and GYY-4137 as Donors of Radioactive Hydrogen Sulfide

Eric M. Brown, James P. Grace, Nimesh P. R. Ranasinghe Arachchige and Ned B. Bowden*

Department of Chemistry, University of Iowa, Iowa City, IA, 52242 United States of America

*Corresponding Author: Ned-Bowden@uiowa.edu

Synthesis of radioactive cysteine trisulfide and glutathione trisulfide. Using the optimized reactions, radioactive Cys-Tris and Glu-TriS were synthesized. A 0.5 mCi solution of $Na_2{}^{35}SO_4$ in 0.5 mL of water was added to 15 mL of degassed HI/NaH_2PO_2 reducing solution. An additional 0.435 g of anhydrous Na_2SO_4 was added. The gas was lead into two washing traps with 15 mL of MilliQ optima water, which lead to the trapping solution of 40 mL of 0.1 M NaOH in MilliQ optima water. Thereafter, it lead to 15 mL of 2 M NaOH and then 15 mL of bleach in order to trap and oxidize any remaining sulfide. The solution was degassed for 10 min, and heated to 130 °C and stirred for 3 h. UV-Vis spectroscopy showed a sulfide concentration of 75.1 mM, giving a yield of 98%.

To the trapped sulfide solution, 1.35 g of $Na_2S \cdot 9H_2O$ was added. In a separate flask, 6.00 g of tributyltin chloride was dissolved in 60 mL of THF and added to the flask containing sulfide. The tributyltin chloride flask was washed with 10 mL of THF and 22 mL of water and added to the reaction flask. The solution was heated to 65 °C and stirred for 16 h. THF was removed under reduced pressure and the aqueous solution was washed 3 times with 20 mL of Et_2O. The organic layers were combined, washed with 15 mL of brine and dried over $MgSO_4$. The solvent was removed under reduced pressure to give bis(tributyltin) sulfide as a colorless oil in a 67% yield. Bis(tributyltin) sulfide was used in the next step without further purification.

To the bis(tributyltin) sulfide, 2.80 g of phthalimide dissolved in 25 mL of DMF was added in one portion. Immediately following, 4.25 g of *N*-bromophthalimide in 20 mL of DMF was added dropwise over 5 min. The solution was stirred for 48 h. The reaction was filtered and washed with 50 mL of toluene yielding MSTR as a white solid in a 46% yield.

The synthesized MSTR (0.427 g) was suspended in 4.7 mL of isopropanol. In a separate flask, 0.412 g of *L*-cysteine was dissolved in 3.5 mL of H_2O. Using a syringe pump, the cysteine solution was added to MSTR with a flow rate of 15 mL/h. Upon full addition of *L*-cysteine, the solution was stirred for an additional 14 h. The solution was filtered and the solid was washed with 30 mL of acetone followed by 30 mL of DCM, affording Cys-TriS as a white solid (625 mg) in a 73% yield.

The remaining MSTR (0.426 g) was suspended in 9.5 mL of isopropanol. In a separate flask, 0.807 g of glutathione was dissolved in 9.5 mL of H_2O. Using a syringe pump, the glutathione solution was added to MSTR with a flow rate of 15 mL/h. Upon full addition of glutathione, the solution was stirred for an additional 25 min. The solution was filtered and the solid was washed with 30 mL of acetone followed by 30 mL of DCM, affording Glu-TriS as a white solid (333 mg) in a 75% yield.

Synthesis of radioactive GYY-4137. Radioactive GYY-4137 was synthesized using the optimized reactions. A 0.5 mCi solution of $Na_2{}^{35}SO_4$ in 0.5 mL of water was added to 12 mL of degassed HI/NaH_2PO_2 reducing solution. An additional 0.305 g of anhydrous Na_2SO_4 was added. The gas was lead into two washing traps with 15 mL of MilliQ optima water, which lead to the trapping solution of 40 mL of 0.1 M NaOH in MilliQ optima water. Thereafter, it lead to 15 mL of 2 M NaOH and then 15 mL of bleach in order to trap any remaining sulfide. The

solution was degassed for 10 min, and heated to 130 °C and stirred for 3 h. UV-Vis spectroscopy showed a sulfide concentration of 52.1 mM, giving a yield of 95%.

The trapped sulfide solution was added to 1.57 g of GYY-Cl in 30 mL of ethyl acetate. 0.770 g of $Na_2S \cdot 9H_2O$ and 3.15 g of tetrabutylammonium bromide were added, and the solution was stirred at 40 °C and monitored by ^{31}P NMR spectroscopy. The reaction was 70% complete after 6 h with the other byproduct being unreacted GYY-Cl. Additional 0.500 g of $Na_2S \cdot 9H_2O$ was added and the reaction was stirred at room temperature for an additional 16 h. The ethyl acetate layer was extracted and the aqueous layer was washed 3x with 20 mL of ethyl acetate. The organic layers were combined and dried over Na_2SO_4 and concentrated under reduced pressure to give the tetrabutylammonium salt of GYY-4137 as an off white solid (1.80 g) in a 65% yield.

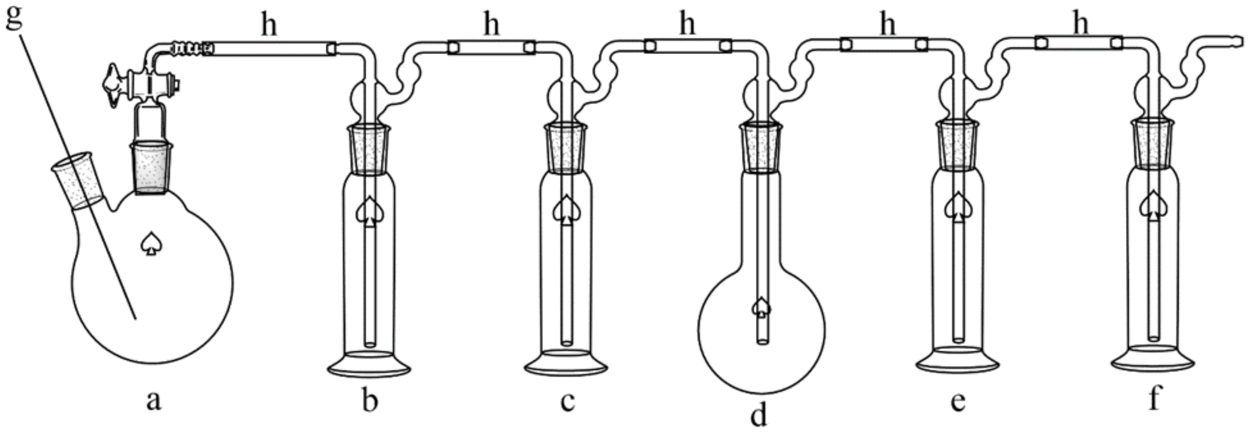

Figure S1. A schematic of the reduction of Na_2SO_4 to H_2S is shown. Teflon tubing (g, 1/16" ID) was pierced through a rubber stopper to bubble the N_2 carrier gas (3 psi) into a 25 mL 2-neck reaction flask (a) that was heated to 130 °C and contained 15 mL 57% HI, 2.0 g NaH_2PO_2, and 450 mg Na_2SO_4. One end of the reaction flask was connected to the Teflon tubing (h, 1/4" ID) and also to two 40 mL Schlenk bubblers (b and c) in series that each contained 15 mL MilliQ optima grade water. The second bubbler was connected to a 50 mL round bottom trapping flask (d) equipped with a gas bubbler adapter and that contained 40 mL of 0.1 M NaOH. Finally, the round bottom flask was connected to two 40 mL Schlenk bubblers containing 15 mL 2 M NaOH (e) and 15 mL bleach (f) using tygon tubing (h).

Table S1. The attempts to precipitate H_2S in organic solvents after the sulfate was reduced are shown.

Trapping Solvent	Trapping Additive	Total Runtime	Notes
Hexanes	NEt_3	30 min	No Precip.
THF	NEt_3	30 min	No Precip.
DMF	NEt_3	30 min	No Precip.<br>Turned blue
DMF	NEt_3 + *N*-bromophthalimide	30 min – 16 h	No Precip.<br>Turned blue
THF	KO^tBu	30 min	No Precip.
Pure NEt_3	-	30 min	No Precip.
Hexanes	NEt_3	1 h – 16 h	No Precip.
Hexanes	NEt_3	1 h – 16 h	No Precip.

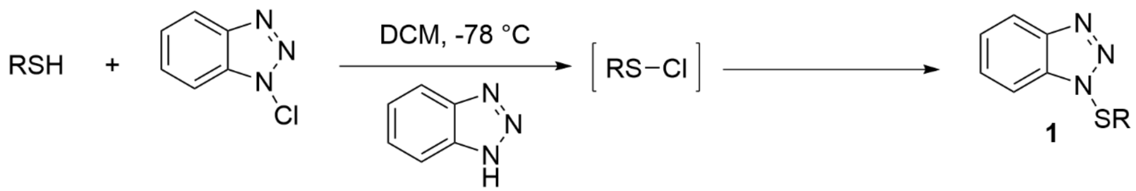

Figure S2. The synthesis of *N*-sulfenyl derivative **1** from the respective thiol, 1-chlorobenzotriazole, and benzotriazole as described by Hunter et al.[1]

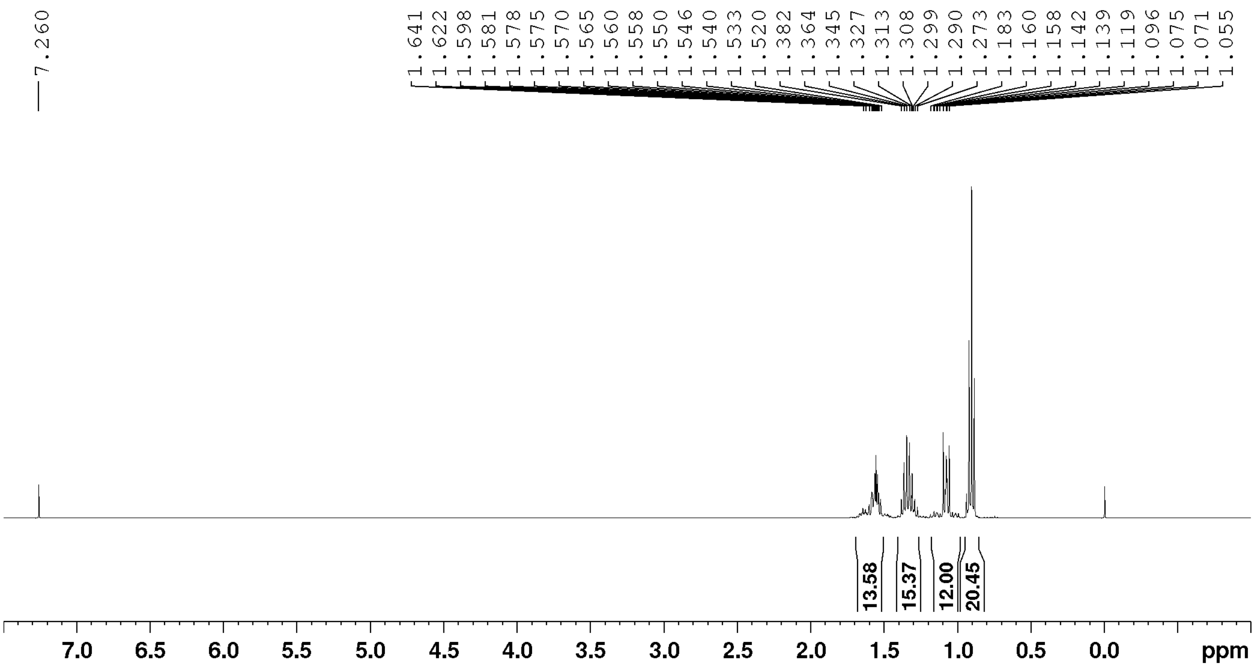
7.260
1.641
1.622
1.598
1.581
1.578
1.575
1.570
1.565
1.560
1.558
1.550
1.546
1.540
1.533
1.520
1.382
1.364
1.345
1.327
1.313
1.308
1.299
1.290
1.273
1.183
1.160
1.158
1.142
1.139
1.119
1.096
1.075
1.071
1.055
13.58
15.37
12.00
20.45
7.0
6.5
6.0
5.5
5.0
4.5
4.0
3.5
3.0
2.5
2.0
1.5
1.0
0.5
0.0
ppm

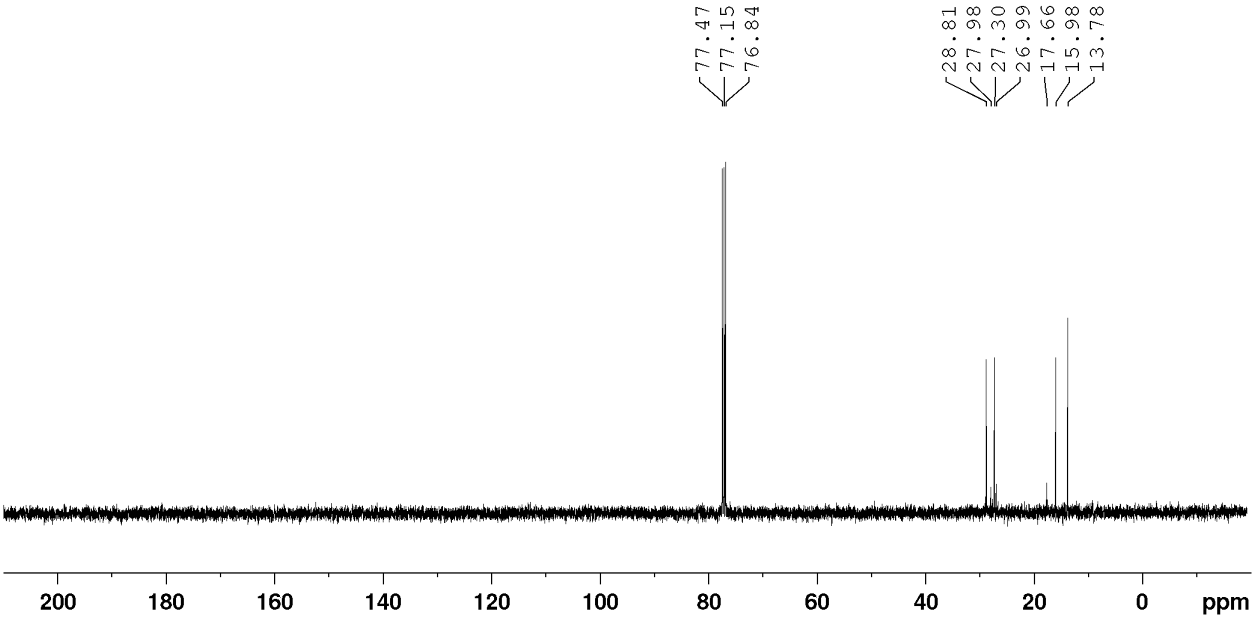
77.47
77.15
76.84
28.81
27.98
27.30
26.99
17.66
15.98
13.78
200
180
160
140
120
100
80
60
40
20
0
ppm

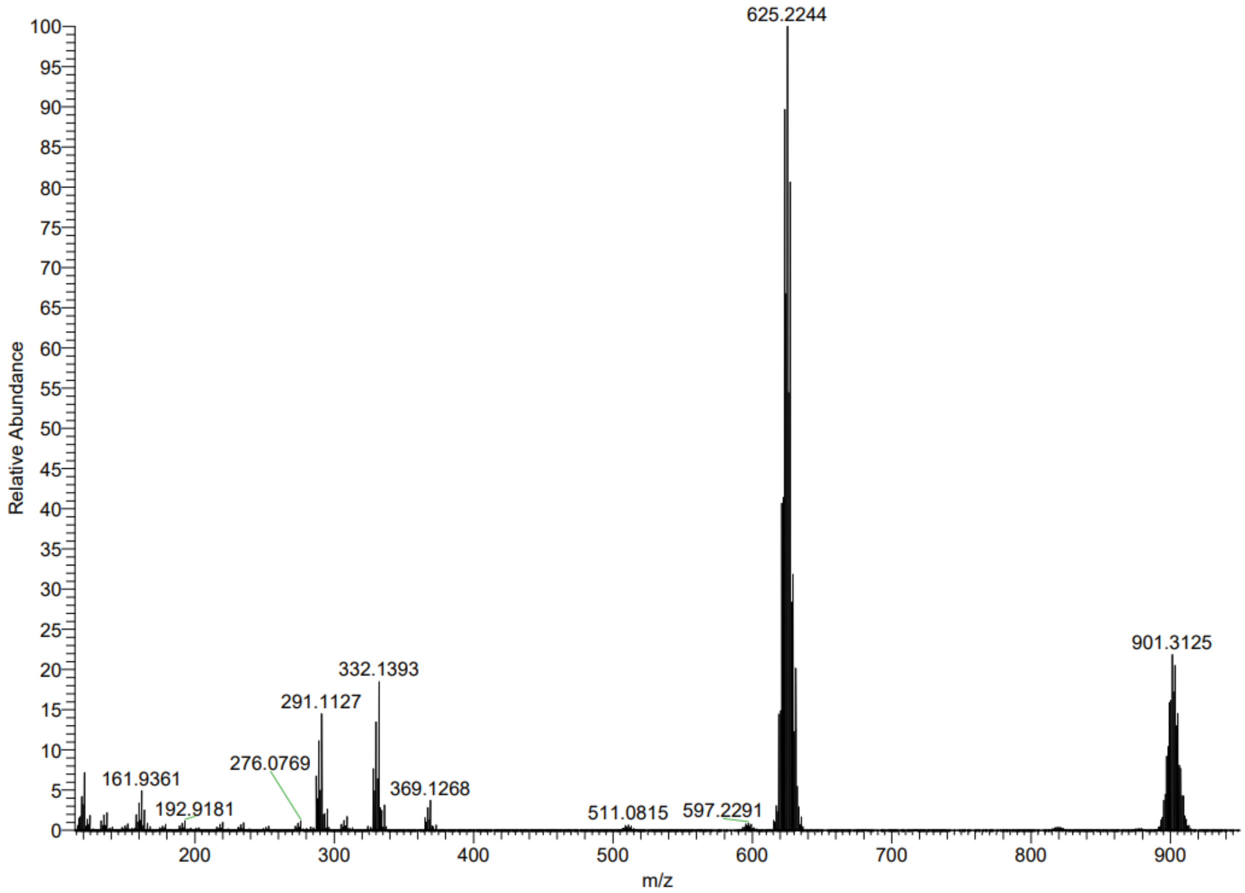

Figure S3. ^{1}H and ^{13}C NMR spectra of bis(tributyltin) sulfide in $CDCl_3$, and HRMS of bis(tributyltin) sulfide.

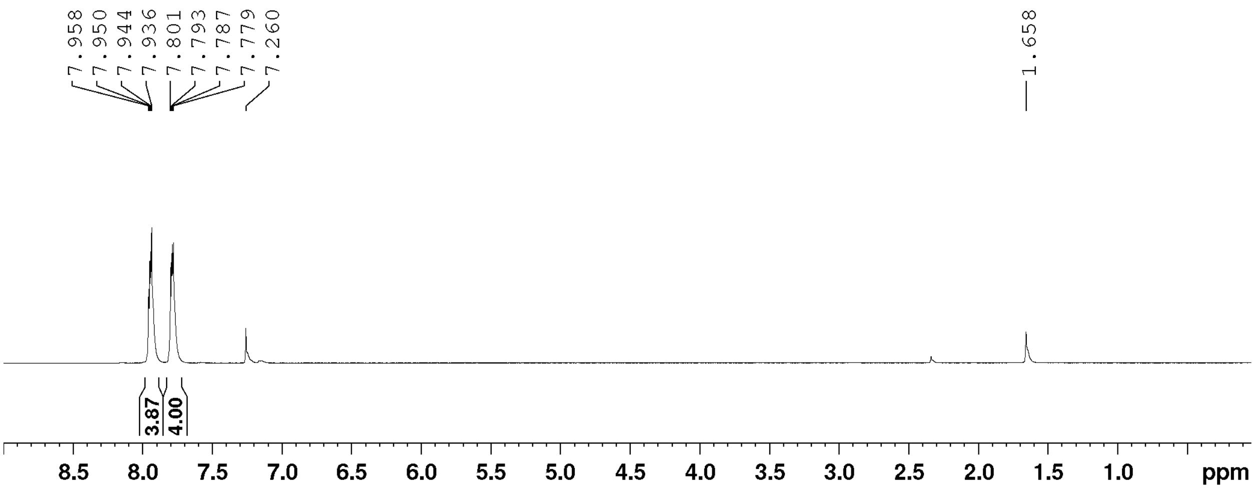
7.958
7.950
7.944
7.936
7.801
7.793
7.787
7.779
7.260
1.658
3.87
4.00
8.5 8.0 7.5 7.0 6.5 6.0 5.5 5.0 4.5 4.0 3.5 3.0 2.5 2.0 1.5 1.0 ppm

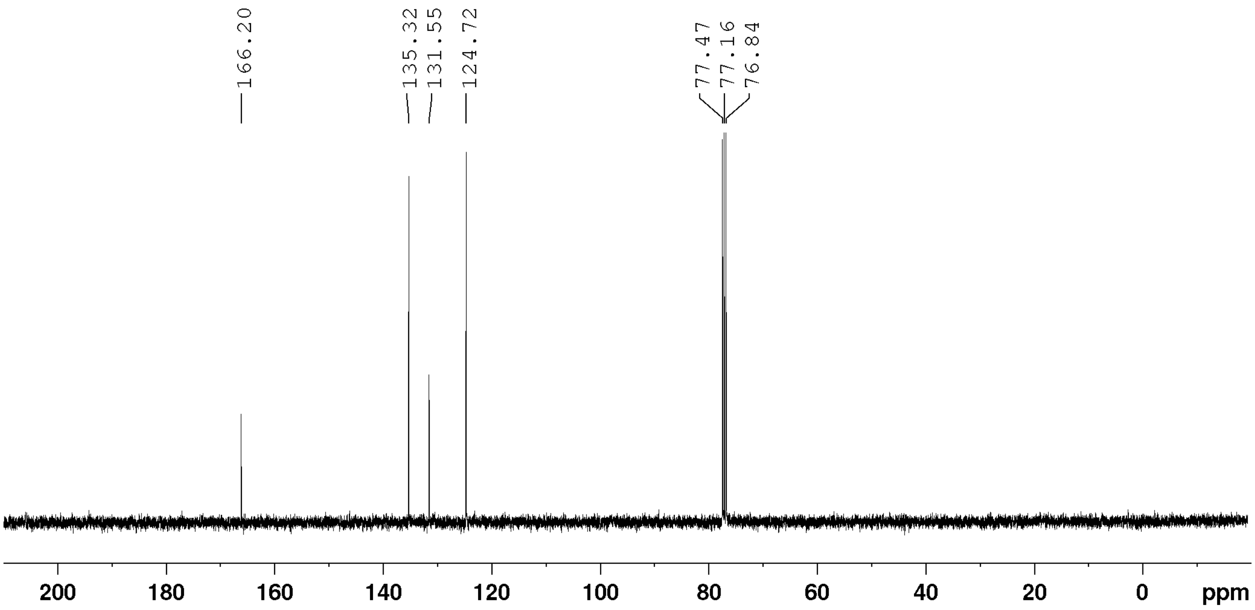
166.20
135.32
131.55
124.72
77.47
77.16
76.84
200 180 160 140 120 100 80 60 40 20 0 ppm

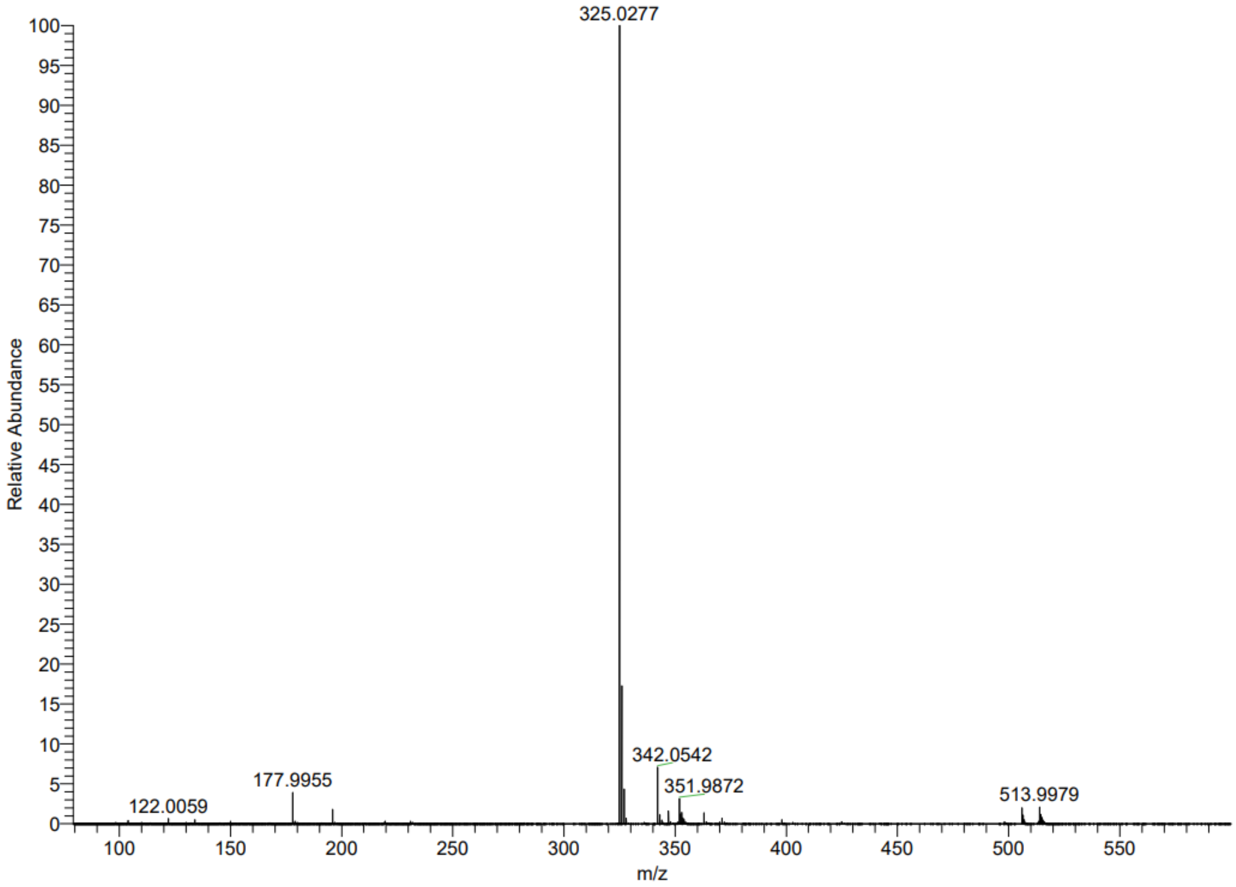

Figure S4. ^{1}H and ^{13}C NMR spectra of 2,2'-thiobis(isoindoline-1,3-dione) (MSTR) in $CDCl_3$, and HRMS of 2,2'-thiobis(isoindoline-1,3-dione) (MSTR).

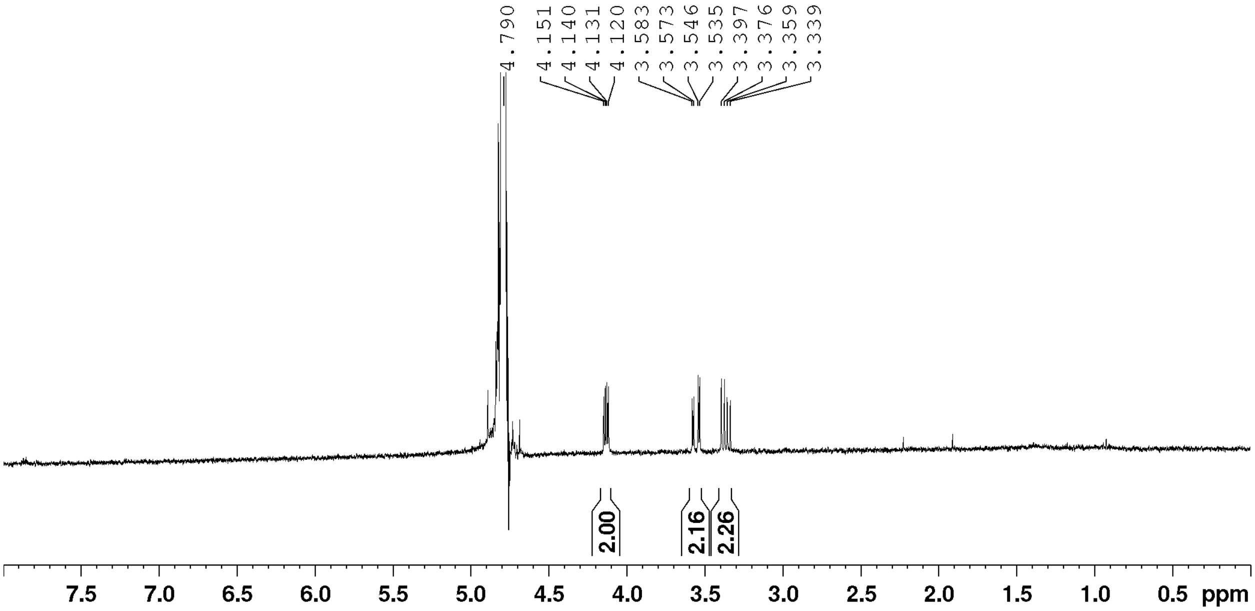
4.790
4.151
4.140
4.131
4.120
3.583
3.573
3.546
3.535
3.397
3.376
3.359
3.339
2.00
2.16
2.26
7.5
7.0
6.5
6.0
5.5
5.0
4.5
4.0
3.5
3.0
2.5
2.0
1.5
1.0
0.5
ppm

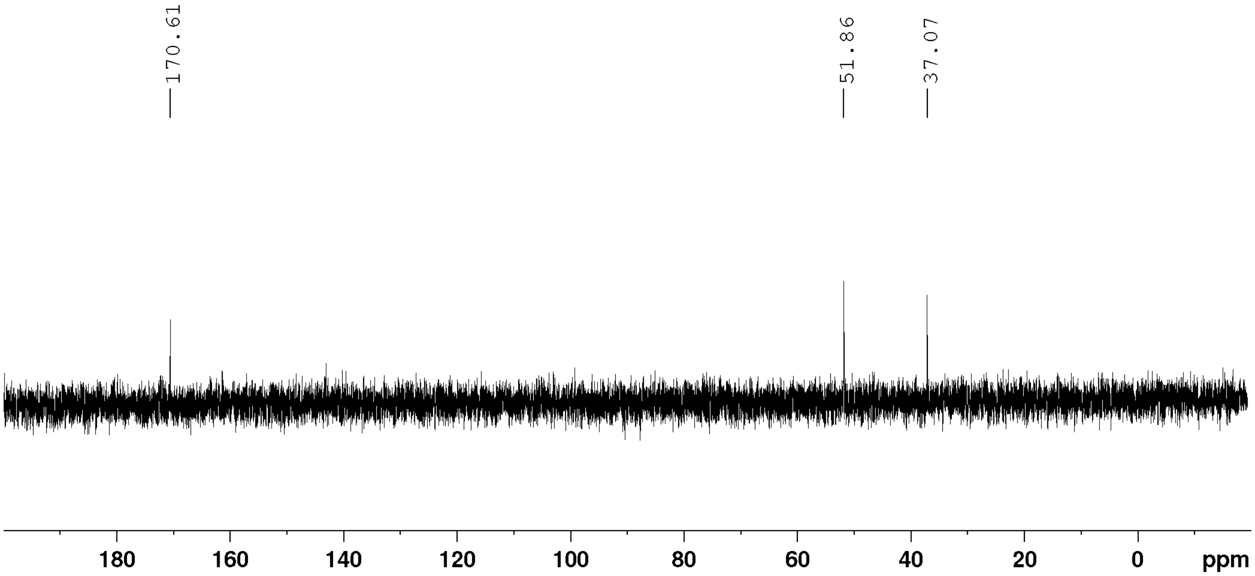
170.61
51.86
37.07
180
160
140
120
100
80
60
40
20
0
ppm

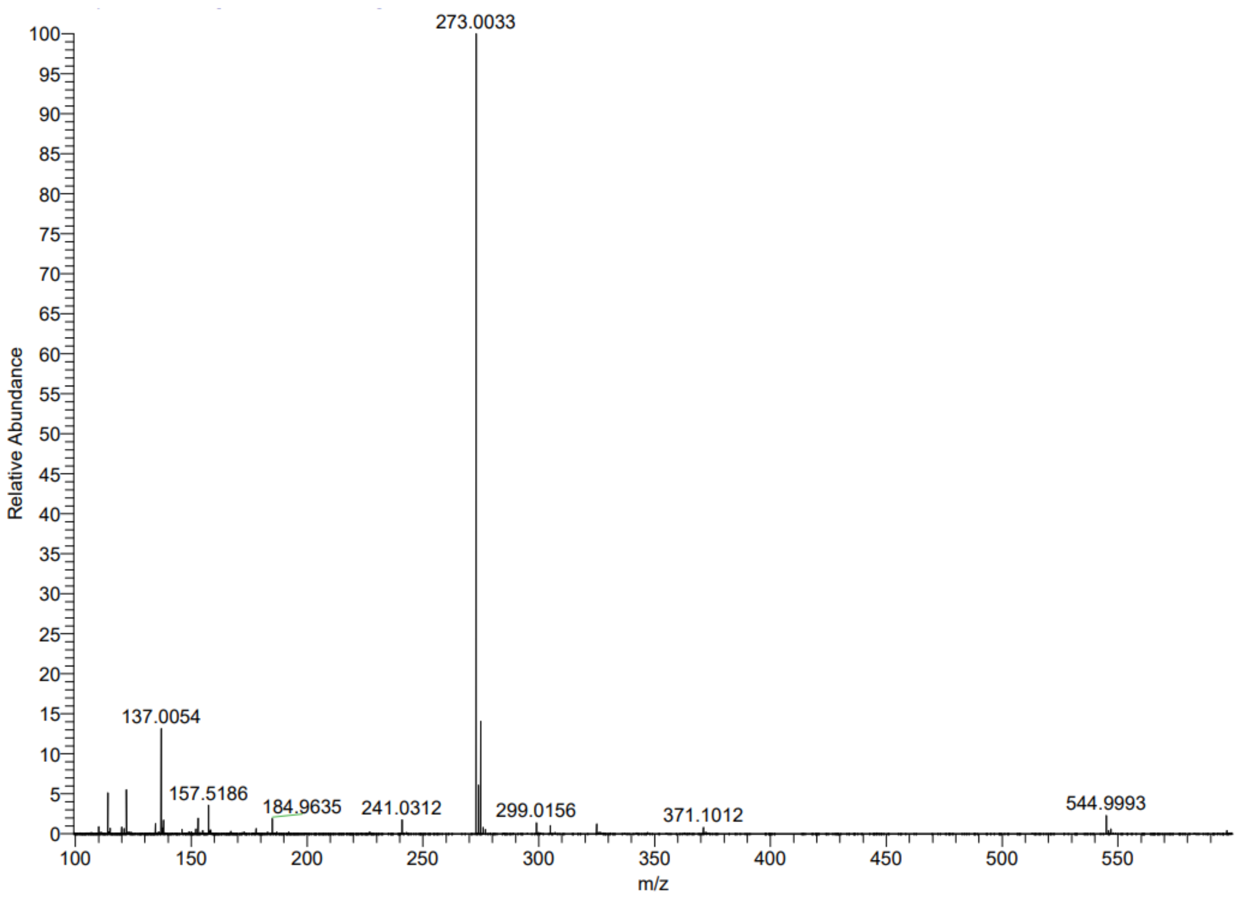

Figure S5. ^{1}H and ^{13}C NMR spectra of cysteine trisulfide in D_2O, and HRMS of cysteine trisulfide.

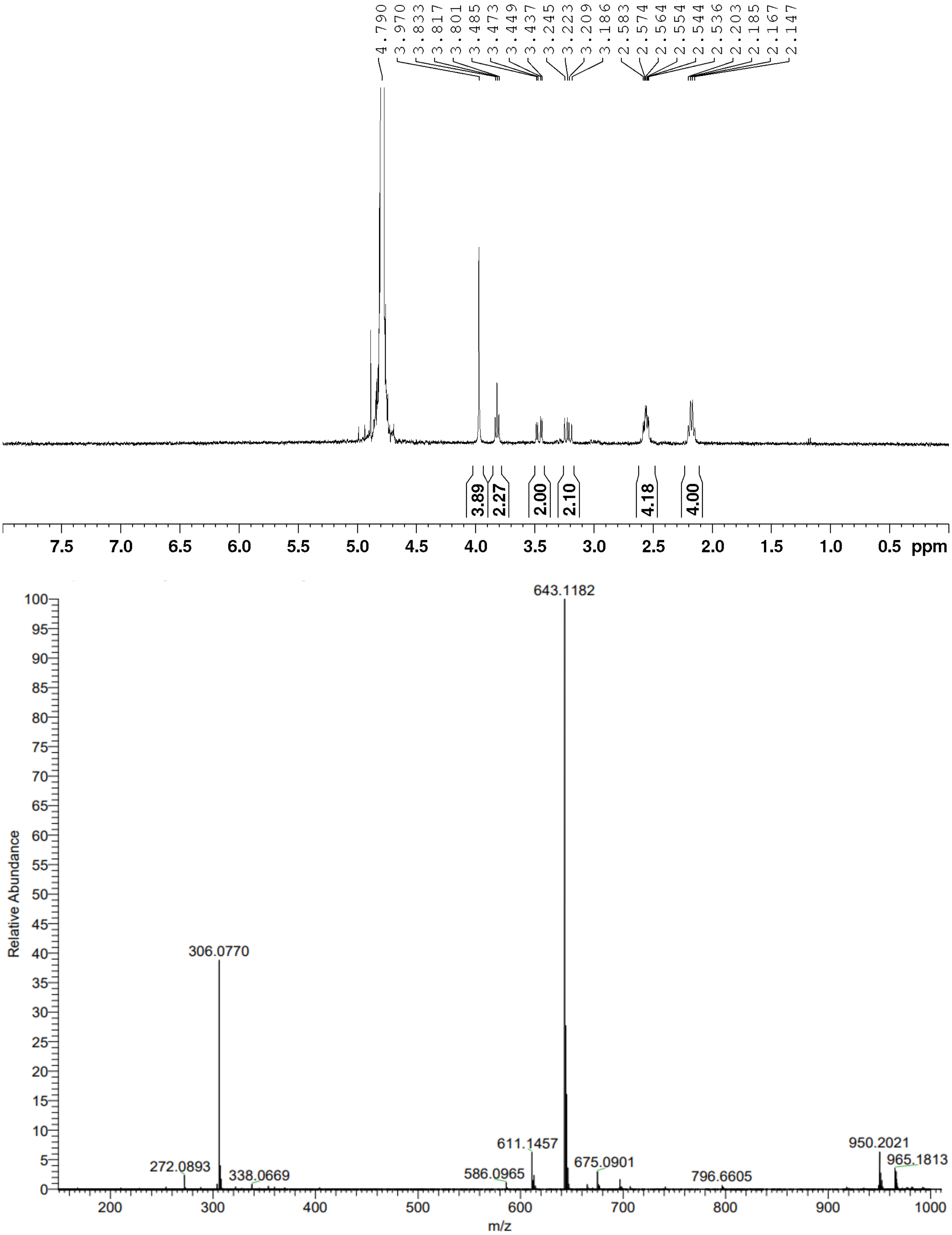

Figure S6. ^{1}H NMR spectrum of glutathione trisulfide in D_2O, and HRMS of glutathione trisulfide.

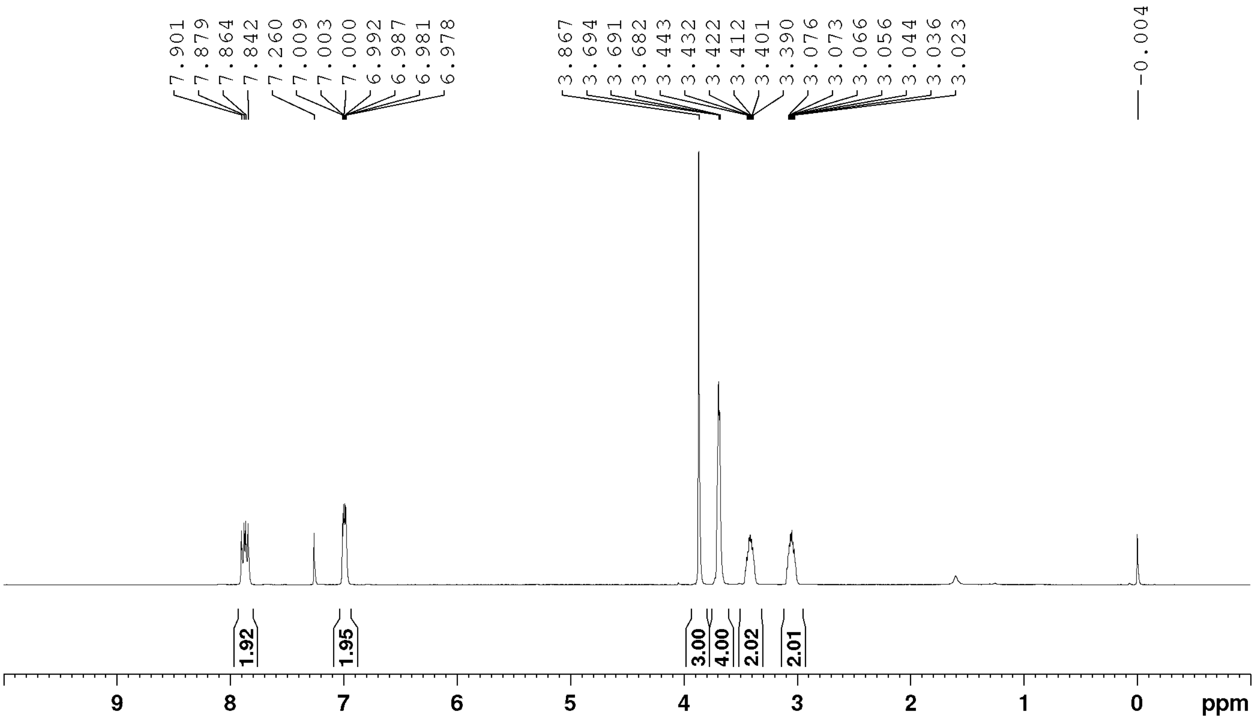
7.901
7.879
7.864
7.842
7.260
7.009
7.003
7.000
6.992
6.987
6.981
6.978
3.867
3.694
3.691
3.682
3.443
3.432
3.422
3.412
3.401
3.390
3.076
3.073
3.066
3.056
3.044
3.036
3.023
-0.004
1.92
1.95
3.00
4.00
2.02
2.01
9 8 7 6 5 4 3 2 1 0 ppm

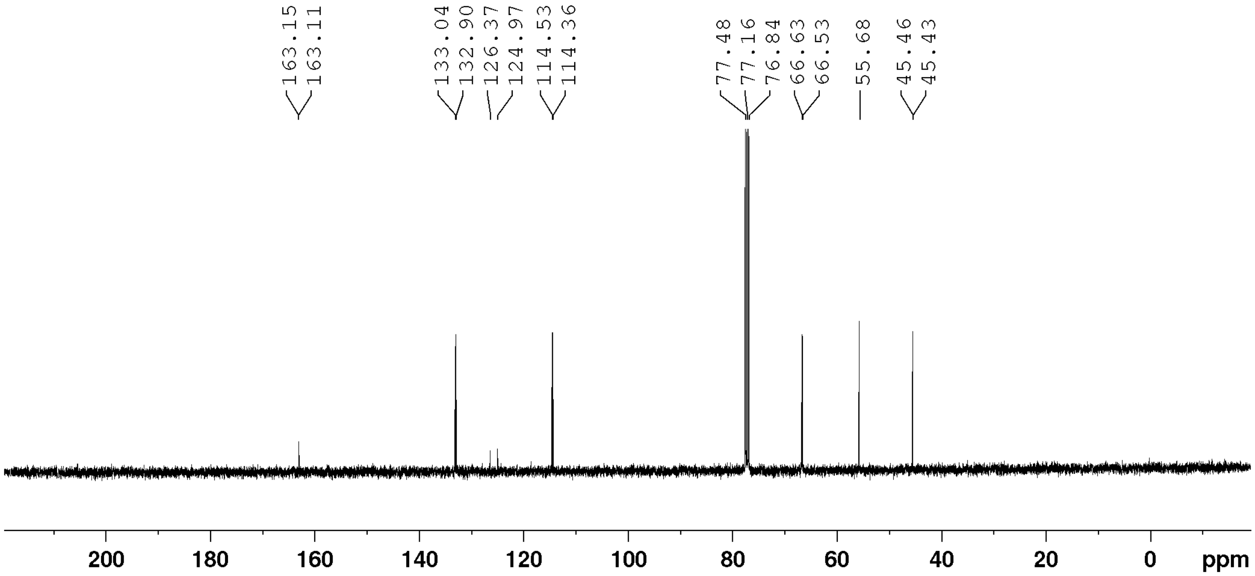
163.15
163.11
133.04
132.90
126.37
124.97
114.53
114.36
77.48
77.16
76.84
66.63
66.53
55.68
45.46
45.43
200 180 160 140 120 100 80 60 40 20 0 ppm

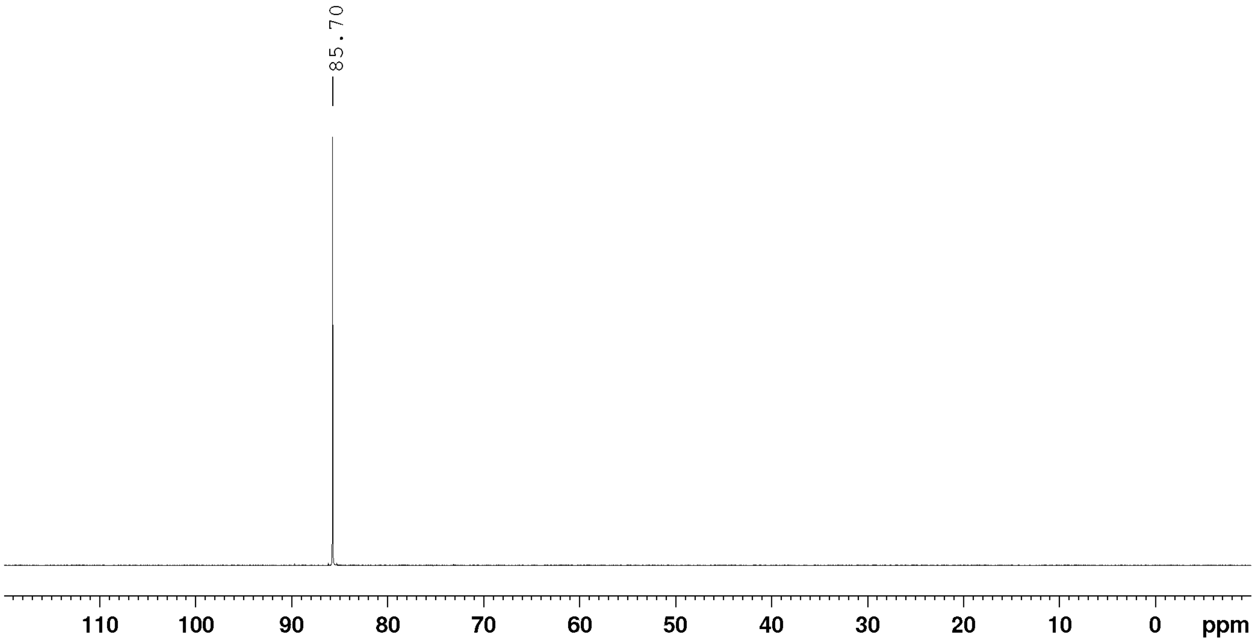

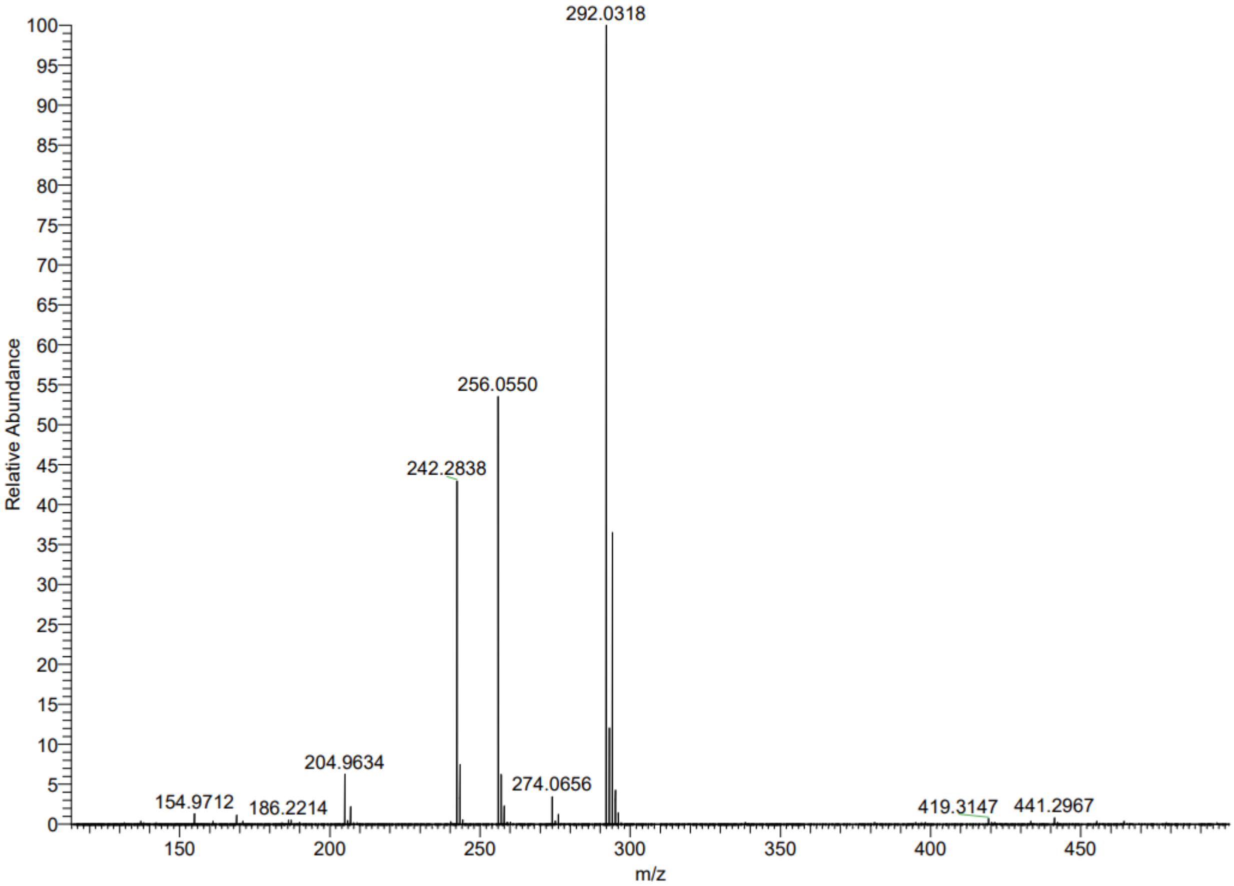

Figure S7. ^{1}H, ^{13}C, and ^{31}P NMR spectra of GYY-Cl in $CDCl_3$, and HRMS of GYY-Cl.

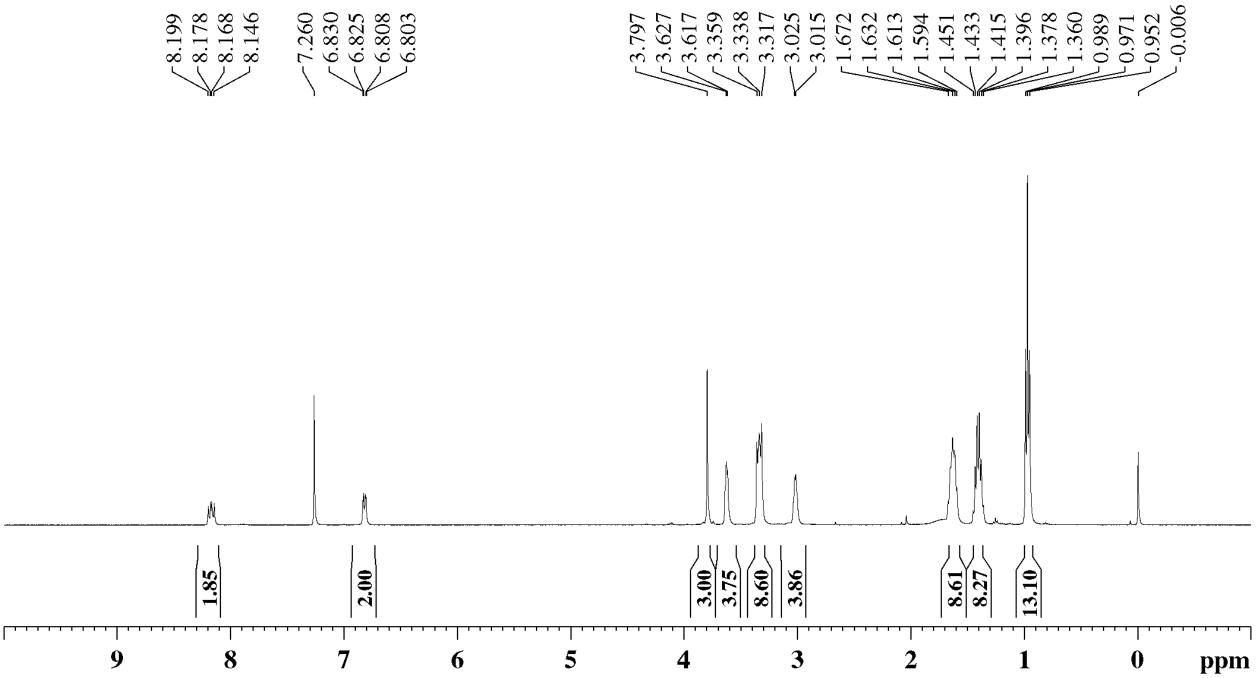
8.199
8.178
8.168
8.146
7.260
6.830
6.825
6.808
6.803
3.797
3.627
3.617
3.359
3.338
3.317
3.025
3.015
1.672
1.632
1.613
1.594
1.451
1.433
1.415
1.396
1.378
1.360
0.989
0.971
0.952
-0.006
1.85
2.00
3.00
3.75
8.60
3.86
8.61
8.27
13.10
9
8
7
6
5
4
3
2
1
0
ppm

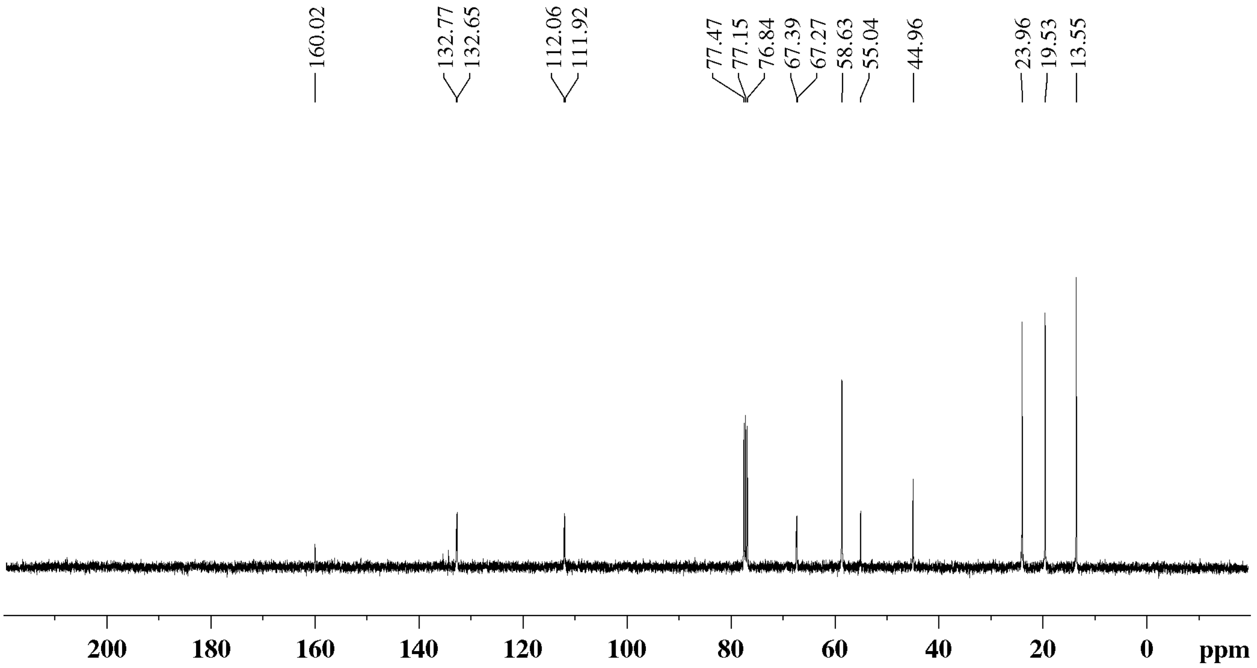
160.02
132.77
132.65
112.06
111.92
77.47
77.15
76.84
67.39
67.27
58.63
55.04
44.96
23.96
19.53
13.55
200
180
160
140
120
100
80
60
40
20
0
ppm

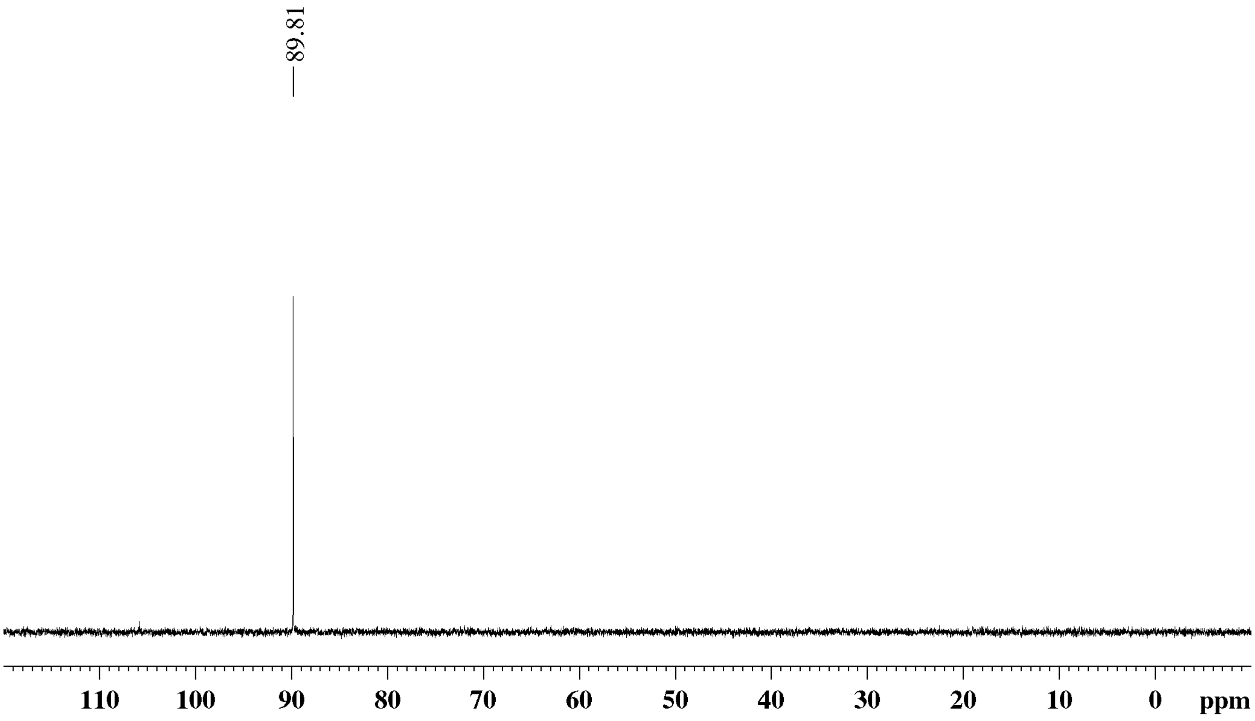

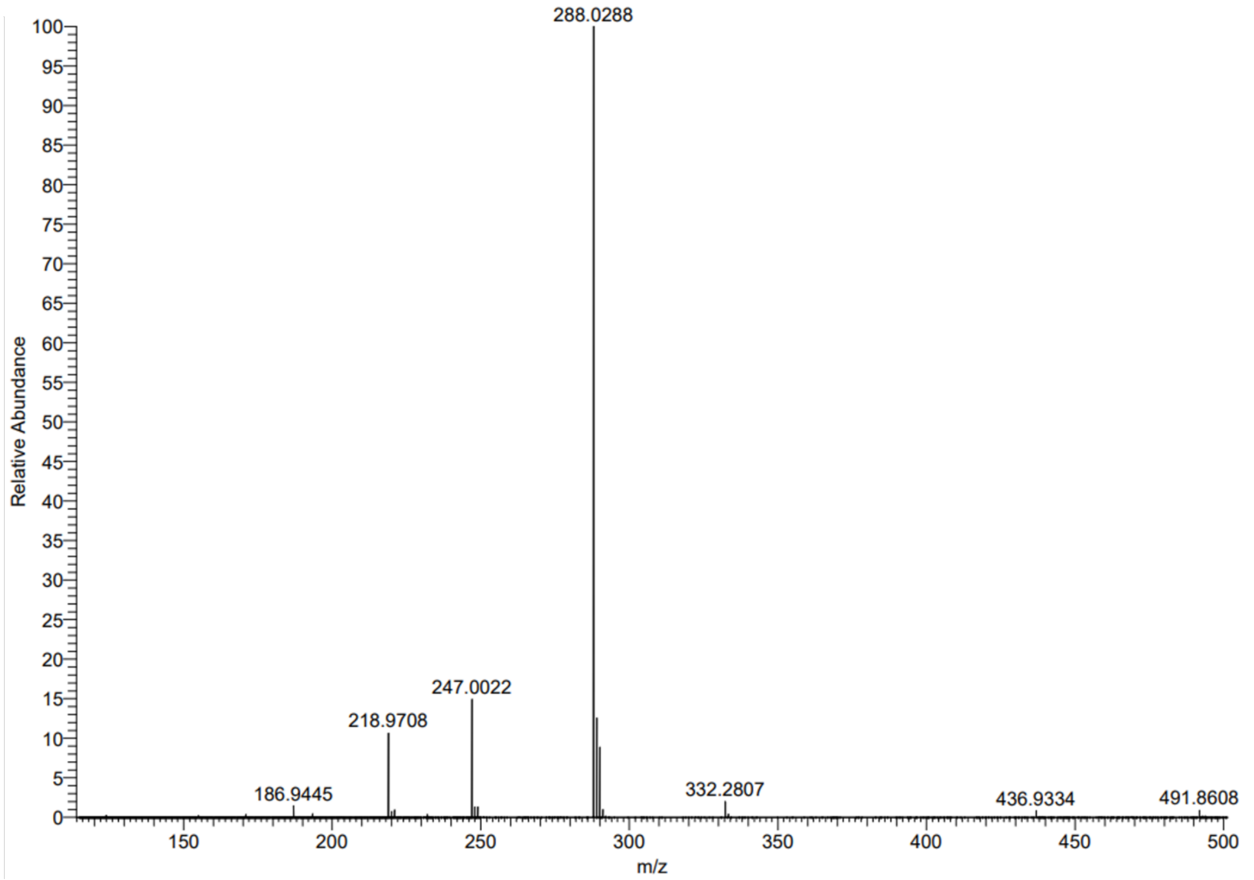

Figure S8. ^{1}H, ^{13}C, and ^{31}P NMR spectra of the tetrabutylammonium salt of GYY-4137 in $CDCl_3$, and HRMS of the tetrabutylammonium salt of GYY-4137.

1. Hunter, R.; Caira, M.; Stellenboom, N. Inexpensive, one-pot synthesis of unsymmetrical disulfides using 1-chlorobenzotriazole. *The Journal of Organic Chemistry* **2006**, *71* (21), 8268-8271.
